# Circular RNA HSDL2 promotes breast cancer progression via miR-7978 ZNF704 axis and regulating hippo signaling pathway

**DOI:** 10.1186/s13058-024-01864-z

**Published:** 2024-06-27

**Authors:** Dandan Wang, Sujin Yang, Mengmeng Lyu, Liping Xu, Shanliang Zhong, Dandan Yu

**Affiliations:** 1 Department of General Surgery, The First Affiliated Hospital of Nanjing Medical University, Guangzhou Road 300, Nanjing, 210029 P.R. China; 2 Department of Gynecologic Oncology, Jiangsu Cancer Hospital and Jiangsu Institute of Cancer Research and the Affiliated Cancer Hospital of Nanjing Medical University, Baiziting 42, Nanjing, 210029 P.R. China; 3 Department of Radiation Oncology, The First Affiliated Hospital of Nanjing Medical University, Guangzhou Road 300, Nanjing, 210029 P.R. China; 4 Center of Clinical Laboratory Science, The Affiliated Cancer Hospital of Nanjing Medical University & Jiangsu Cancer Hospital & Jiangsu Institute of Cancer Research, Baiziting 42, Nanjing, 210009 China

**Keywords:** Breast cancer, Circular RNA, CircHSDL2, miR-7978, Hippo signaling

## Abstract

Circular RNAs (circRNAs) are a new group of endogenous RNAs recently found to be involved in the development of various diseases, including their confirmed involvement in the progression of several types of cancers. Unluckily, the abnormal expression and functions of circRNAs in breast cancer shall be further investigated. This work aims to elucidate the action and molecular mechanism of circHSDL2 in the malignant progression of breast cancer. Differential expression profiles of circRNAs in breast cancer tissues relative to normal breast tissues and in the exosomes of breast cancer patients compared to healthy women were analyzed from databases to identify potentially functional circRNAs. CircHSDL2 was selected for further investigation. Cell proliferation, migration and invasion assays were done to assess the effect of circHSDL2 overexpression on breast cancer cells. Bioinformatics test and dual-luciferase reporter experiments were done to explore the interaction between circHSDL2 and miRNA. Downstream target genes were further investigated through proteomics analysis and Western blotting. The influence of circHSDL2 on breast cancer in vivo was evaluated through xenograft experiments in nude mice. Functional analysis demonstrated circHSDL2 overexpression promoted the division, movement, and invasion of breast cancer cells both in vivo and in vitro. Mechanistically, circHSDL2 acted as a sponge for miR-7978 to affect ZNF704 expression and thereby regulate the Hippo pathway in breast cancer cells. In conclusion, circHSDL2 regulates the Hippo pathway through the miR-7978/ZNF704 axis to facilitate the malignancy of breast cancer. This may be a potential biomarker and treatment target.

**Supplementary Information:**

The online version contains supplementary material available at 10.1186/s13058-024-01864-z.

## Introduction

Breast cancer has the highest incidence rate and its mortality ranks first among women worldwide [1]. Recently, there is a downtrend in the overall mortality rate of breast cancer, but the mortality rate of advanced breast cancer is still stubbornly high [2]. The treatment of advanced breast cancer primarily relies on chemotherapy, radiation therapy, and targeted therapy, but these treatments have suboptimal efficacy and significantly challenge comprehensive breast cancer management. Therefore, exploring the molecular mechanisms of breast cancer invasion and metastasis and identifying new targets related to malignant progression have become crucial approaches in treating advanced breast cancer.

Circular RNAs (circRNAs) are a new class of closed-loop RNA structures primarily formed through exon or intron circularization. CircRNAs differ from typical linear mRNAs or ncRNAs in their structural characteristics, as they possess closed-loop covalent structures and lack both 5’ cap and 3’ poly(A) tails [3]. Due to their resistance against exonuclease degradation and lack of free ends, circRNAs exhibit high cellular stability. With advancements in RNA sequencing technology and bioinformatics, hundreds of circRNAs with developmental regulation, tissue specificity, and subcellular localization have been identified [4, 5]. In recent years, an increasing body of research suggests that circRNAs are dysregulated in various human diseases. The reported functions of circRNAs include competitive miRNA sponges, competitive binding regulators of protein factors, and regulators of peptide transcription and translation [6–8]. Recent research has discovered that circRNAs are critical in tumor emergence, advancement and metastasis, such as circ-ITCH inhibiting cell division by suppressing the Wnt/ß-Catenin pathway in lung cancer, Cdrlas acting as an oncogene by competitively binding to miR-7 in hepatocellular carcinoma and circUHRF1 has an oncogenic effect on oral squamous cell carcinoma tumorigenesis via feedback loop [9–11]. CircRNAs have also been implicated in the treatment of tumors. Zhang et al. found that exosomal circUHRF1 contributed to immunosuppression and may cause resistance to anti-PD1 therapy in hepatocellular cancer [12]. All these studies indicate that circRNAs may be potential biomarkers and targets in cancer treatment. However, since most circRNAs remain incompletely characterized and their roles in breast cancer progression are largely unknown, further research is needed to identify breast-cancer-related circRNAs and their functions.

Herein, we first downloaded and tested the expression profiles of circRNAs in 8 breast cancer tissue samples and 3 normal breast tissue samples from the GEO database. After comparison, we identified totally 399 significantly differentially expressed circRNAs (DECs), including 208 upregulated and 191 downregulated circRNAs. Then we analyzed and selected 676 DECs from the published circRNA expression profiles of serum exosomes from 3 breast cancer patients and 3 healthy volunteers, including 652 upregulated and 24 downregulated circRNAs [13]. We took the intersection of DECs from exosomes and tissue samples, resulting in 3 commonly upregulated circRNAs (circ-0088088, -0005455, -0008016) and 0 commonly downregulated circRNA. We selected circ-0088088 for further investigation. The source gene of circ-0088088 is HSDL2, which was referred to as circHSDL2. Results showed circHSDL2 overexpression significantly promoted the division, motion and invasion of breast cancer cells. Importantly, we explored the mechanism of circHSDL2 in breast cancer progression and found it acted as a sponge of miR-7978, releasing miR-7978 inhibition of ZNF704 and hence aberrantly regulating the Hippo pathway. Our research elucidates a novel mechanism of malignant progression in breast cancer and offers new insight into the diagnosis and therapy of breast cancer.

## Materials and methods

### Cells culture

Human breast cancer cell line MDA-MB-231 was offered by the Cell Bank of Chinese Academy of Sciences (Shanghai, China). Cells were cultivated at 37 ℃ with 5% CO_2_ in high-glucose Dulbecco’s modified Eagle’s medium (DMEM) (Gibco, Carlsbad, CA, USA) added with 10% fetal bovine serum (FBS) (Gibco), 100 ug/ml penicillin and 100 U/ml streptomycin.

### Fluorescence in situ hybridization (FISH)

The specific probes for circHSDL2 labeled with Cy3 were obtained by RiboBio (Guangzhou, China). The Ribo™ FISH kit (RiboBio) was operated as instructed by the manufacturer. CircHSDL2 was hybridized using Cy3-labeled probe. Cell nucleus was stained with 4,6-diamidino-2-phenylindole (DAPI) [11]. A confocal microscope (Carl Zeiss, Germany) was used for observation and photo taking.

### CircRNA lentivirus construction and transfection

A lentivirus carrying hsa-circHSDL2 and luciferase was constructed to establish a stable cell line (Hanbio, Shanghai, China). Cells infected with the lentivirus were selected with 2.5 ug/ml puromycin.

The miRNA mimics, siRNA and negative control were bought from RiboBio. MiRNA mimics negative control contains a sequence with no known targets. It does not bind to any known mRNA targets and does not induce any specific gene regulation changes within the cell. MDA-MB-231 cells were transfected with a Lipofectamine 8000 reagent (Beyotime, Shanghai, China) as per relevant instructions.

### RNA extraction and quantitative real-time polymerase chain reaction (RT-qPCR)

Total RNA of cells was isolated with a Trizol reagent (Invitrogen, CA, USA). Then RNA content and purity were measured with a Nanodrop 2000 spectrophotometer (Thermo Fisher Scientific, USA). cDNA for circHSDL2 and miRNAs was synthesized with a PrimeScript™ RT master mix kit and a Mir-X™ miRNA first-strand preparation kit (both Takara, Japan). RT-qPCR was conducted with Taq pro universal SYBR qPCR master mix (Vazyme Biotech, China). The primers were obtained by Sangon Biotech (Shanghai, China) and listed together with siRNAs in Table S1. Relative expressions of circHSDL2 and miRNAs were analyzed with the 2^−△△Ct^ approach.

### Cell proliferation assay

The proliferating activity of the breast cancer cells was evaluated via EdU staining. Cells were grown in a medium with a 50 µM EdU reagent (RiboBio) for 2 h and fixed with 4% paraformaldehyde. Then they were stained with 1×Apollo^®^ dyeing solution. The nuclei were stained with Hoechst33342. Datas were captured and observed under an inverted fluorescent microscope (Olympus, Japan).

### Wound healing and transwell assay

Cells were planted in a 6-well plate until reaching 90% confluence and scratched with a 200 µL pipette tip in the middle of the plate. The cells were further cultivated in a serum-free medium. Cell migration was observed with the inverted microscope at both 0 and 48 h.

Cell migration and invasion assays were done with Transwell chambers (Corning, USA) without and with Matrigel (BD Science, USA) respectively. About 2 × 10^4^ cells were suspended in 200 µL of FBS-free DMEM and seeded into the upper chambers. Then 600 µL of DMEM with 20% FBS was added into the lower chamber. After 24 h, the migrating or invading cells were fixed with 4% paraformaldehyde and dyed with crystal violet. Pictures were captured with the inverted microscope.

### Dual-luciferase reporter assay

Dual-luciferase reporter assays were carried out as previously described [14]. The sequences of circHSDL2 and the mutant version without miR-7978 binding sites were amplified and cloned into a luciferase reporter vector pmiR-RB-Report™ (RiboBio), termed circHSDL2-wt and circHSDL2-mut respectively. HEK293T cells (1.5 × 10^4^) were planted into 96-well plates and cotransfected with these plasmids and mimics or the control. Then the firefly and Renilla luciferase activity was tested using a dual-luciferase assay kit as per relevant protocols (Beyotime).

### MiRNA and mRNA forecasting

The target miRNAs that may bind to circHSDL2 were predicted with CircMir (http://www.bioinf.com.cn/) and the Cancer-Specific CircRNA Database (CSCD, http://gb.whu.edu.cn/CSCD/). CircMir is based on miRanda 2010 Release (http://www.microrna.org/microrna/getDownloads.do) and RNAhybrid-2.1.2 (https://bibiserv.cebitec.uni-bielefeld.de/rnahybrid/) to predict circRNA-miRNA interactions. The intersection of miRNAs predicted by the three databases was used for further study.

Databases of miRDB (https://mirdb.org/), miRtarbase (https://mirtarbase.cuhk.edu.cn/), and targetscan (http://www.targetscan.org/) were used to forecast the target mRNAs of miR-7978. The target mRNAs were further intersected with upregulated proteins in the proteomics results of MDA-MB-231 cells overexpressing circHSDL2.

### Label-free quantitative proteomics

Proteomics analysis of breast cancer cells was conducted by Biotree (Shanghai, China). MDA-MB-231 cells overexpressing circHSDL2 or the control were made for LC-MS as required by Biotree. In brief, samples were prepared by protein extraction, protein digestion and peptide desalting. Then total peptides of the samples were isolated and tested with nano-UPLC (EASY-nLC1200) connected to a Q-Exactive HFX Orbitrap device (Thermo Fisher Scientific) with nano-electrospray. A reversed-phase column (100 μm ID × 15 cm, ReprosilPur 120 C18AQ, 1.9 μm, Dr. Maisch, Germany) was used in the separation. All procedures were finished in Biotree.

### Western blotting

Breast cancer cells were ice-lysed with an RIPA buffer. Proteins were extracted after centrifugation and quantified with a BCA kit (Biosharp, China). Same quantities of proteins (30 µg) were electrophoresed on 10% SDS-PAGE and transferred to PVDF membranes (Millipore, USA). After blocking for 2 h with 5% skim milk, the membranes were immunoblotted with primary antibodies at 4 ℃ overnight, including anti-ZNF704 (Santa Cruz, #SC-514,109, USA), anti-MST2/Krs-1 (Santa Cruz, #SC-130,405), anti-YAP1 (Abcam, #ab52771, UK), anti-YAP1 (phosphor S127) (Abcam, #ab76252), anti-LATS1/2 (Affinity, #DF7517, USA), anti-phospho-LATS1/2 (Ser909/Ser872) (Affinity, #AF8163), anti-TAZ (Cell Signaling Technology, #72,804, USA) and anti-GAPDH (Proteintech, USA). The membranes were cultured with a secondary antibody (Beyotime) for 1 h after washing with TBST and visualized using an enhanced chemiluminescence (ECL) plus kit (Millipore) and an integrated chemiluminescence Datar (CLINX, ChemiScope 5300 Pro, China). The Datas were tested on Data J.

### Animal experiments

Approval was offered by the Animal Care and Use Committee of Nanjing Medical University. Athymic nude mice (female BALB/c, 4 to 6 weeks old) were bought from Beijing Vital River Laboratory Animal Technology Co., Ltd. The MDA-MB-231 cells stably expressing circHSDL2 or the control (5 × 10^6^ cells/mouse) were injected subcutaneously into the right flank (*n* = 5, each group) to build a subcutaneous implantation model. Tumor length and width were detected every 2 days. Tumor volumes were estimated as volume (mm^3^) = 0.5 * length * witdth^2^. The tumors were weighed at the end point of the assay. For lung metastasis assay, MDA-MB-231 cells stably expressing circHSDL2 or the control were luciferase-labeled, and injected into the caudal vein. Lung metastasis was detected using a visible light 3D imaging system for animals (PerkinElmer, IVIS Spectrum, USA).

### Immunohistochemistry

Fresh samples of xenograft tumor tissues were fixed with formalin, dehydrated with gradient aqueous alcohol, embedded with paraffin and cut into 4-µm sections. Then the sections were grown with anti-ZNF704, anti-TAZ and anti-MST2/STK3 (Abcam, ab52641). Pictures were taken under the inverted microscope.

### Statistical analysis

Statistical analysis was finished on GraphPad Prism 7 (GraphPad, CA, USA) and SPSS 20.0 (IBM, SPSS, IL, USA). Groups were compared via Student’s t test. Data were acquired from three independent assays and expressed as mean ± standard deviation (SD). *P* < 0.05 implied significance.

## Results

### Identification and characterization of circHSDL2 in breast cancer cells

**Fig. 1:**
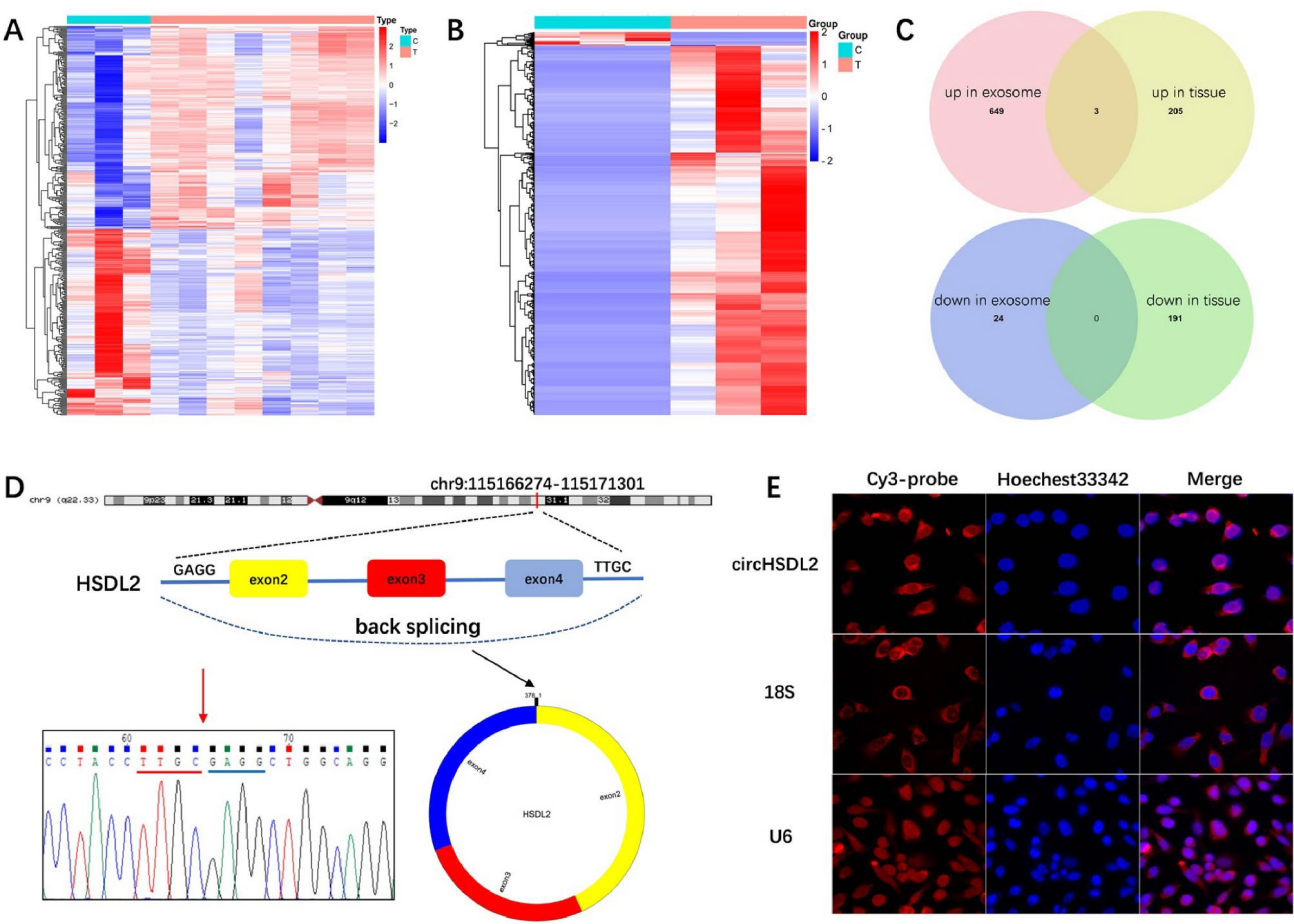
Recognition and characterization of circHSDL2 in breast cancer cells. **(A)** Expression profiles of differentially expressed circRNAs (DECs) in 8 breast cancer tissue samples versus 3 mammary gland tissue samples from GEO. **(B)** Expression profiles of DECs of human serum exosomes from 3 BCa patients versus 3 healthy volunteers. **(C)** Intersection of DECs in exosomes and tissues. **(D)** Genomic loci of the HSDL2 gene and circHSDL2. The red arrow indicates the sequences of the reverse splicing regions of exons 2 and 4, which were validated by Sanger sequencing. **(E)** FIsh assay shows circHSDL2 is primarily located in the cytoplasm. The circHSDL2 probe was marked with Cy3 (in red), and the cell nuclei were dyed with Hochest 33,342 (in blue)

We firstly downloaded circRNA datasets (GSE101123, analyzed on Agilent-069978 Arraystar Human CircRNA microarray V1), including circRNA expression in 8 breast cancer tissue samples and 3 mammary gland tissue samples from GEO (https://www.ncbi.nlm.nih.gov/geo/). Totally 399 differentially expressed circRNAs (DECs) (208 in upregulation and 191 in downregulation) were screened out at fold change (FC) > 1 and *p* < 0.05 using Limma of R/Bioconductor (Fig. 1A). Then the published RNA-seq data of human serum exosomes from 3 BCa patients and 3 controls were obtained [13]. A total of 676 DECs (652 in upregulation and 24 in downregulation) were obtained using Limma with thresholds of absolute value of log (FC) > 1 and adj. *p* < 0.05 (Fig. 1B). The intersections of DECs in exosomes and tissues were shown in Venn diagrams (Fig. 1C). We got 3 co-upregulated circRNAs (circ-0088088, -0005455 and − 0008016) and 0 co-downregulated circRNA. In the previous research of our team, we confirmed that the expression of circ-0088088 in serum exosomes of eight breast cancer patients was significantly higher than that in the serum exosomes of eight healthy individuals [15]. We considered that circ-0088088 was likely involved in the malignant progression of breast cancer and selected circ-0088088 for further study.

The source gene of circ-0088088 is HSDL2, so we call circ-0088088 as circHSDL2. CircHSDL2 is 378 bp long and formed by circularizing exon 2–4 of the gene HSDL2. It is positioned at chr9:115166274–115,171,301. The back splicing junction of circHSDL2 was verified via sanger sequencing (Fig. 1D). FISH mainly identified circHSDL2 in the cytoplasm (Fig. 1E).

### CircHSDL2 contributes to proliferation, migration and invasion of BCa cells

**Fig. 2:**
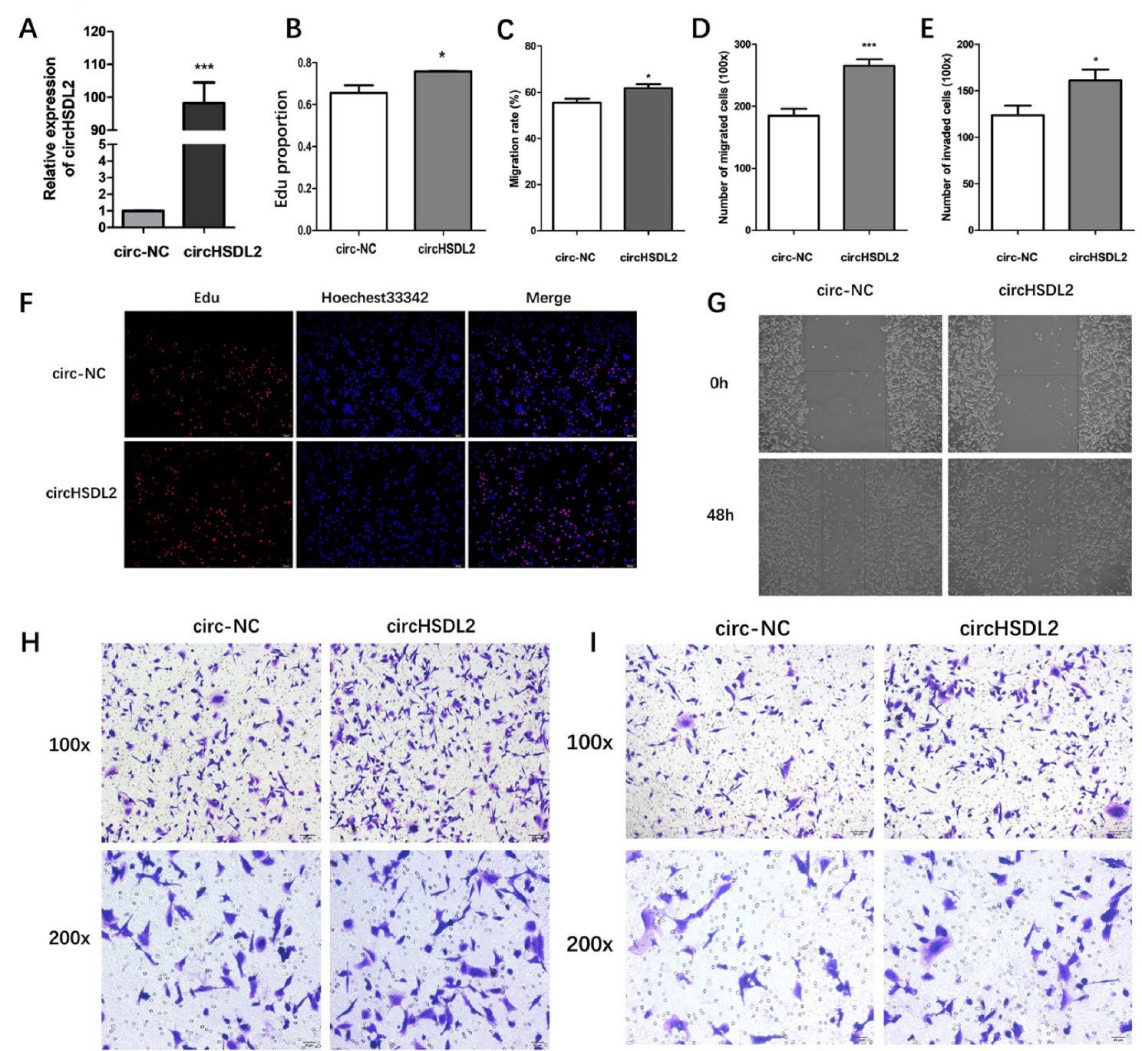
CircHSDL2 promotes proliferation, migration and invasion of BCa cells. **(A)** Relative expression of circHSDL2 in MDA-MB-231 cells after stable transfection with circHSDL2 lentivirus or negative control. **(B**, **F**) EdU assay for proliferation capacity and (**C**, **G**) wound healing assay for migration ability of breast cancer cells transfected with circHSDL2 or negative control. (**D**, **E**, **H**, and **I**) Impact of circHSDL2 or negative control transfection on the motion and invasion of breast cancer cells evaluated through transwell and matrigel assays. Data were presented as mean ± SD from at least three independent assays, *p < 0.05, **P < 0.01, ***P < 0.001

To probe into the potential roles of circHSDL2 in BCa cells, we established circHSDL2 cells stably overexpressing MDA-MB-231 via transduction with lentivirus. The qRT-PCR revealed the overexpression ability of circHSDL2 (Fig. 2A). EdU assays implied the percent of EdU-positive cells in overexpressing circHSDL2 BCa cells was remarkably higher than in the control group (Fig. 2B, F). This result indicates circHSDL2 promotes BCa cell proliferation. Wound healing assay displayed circHSDL2 overexpression increased the migrating rate of BCa cells (Fig. 2C, G). Transwell migration and Matrigel invasion assays exhibited that circHSDL2 overexpression contributed to the migration and invasion of BCa cells (Fig. 2D, E, H, I). These results suggest circHSDL2 promotes the division, movement and invasion of BCa cells.

### CircHSDL2 as a sponge for miR-7978 in BCa cells

**Fig. 3:**
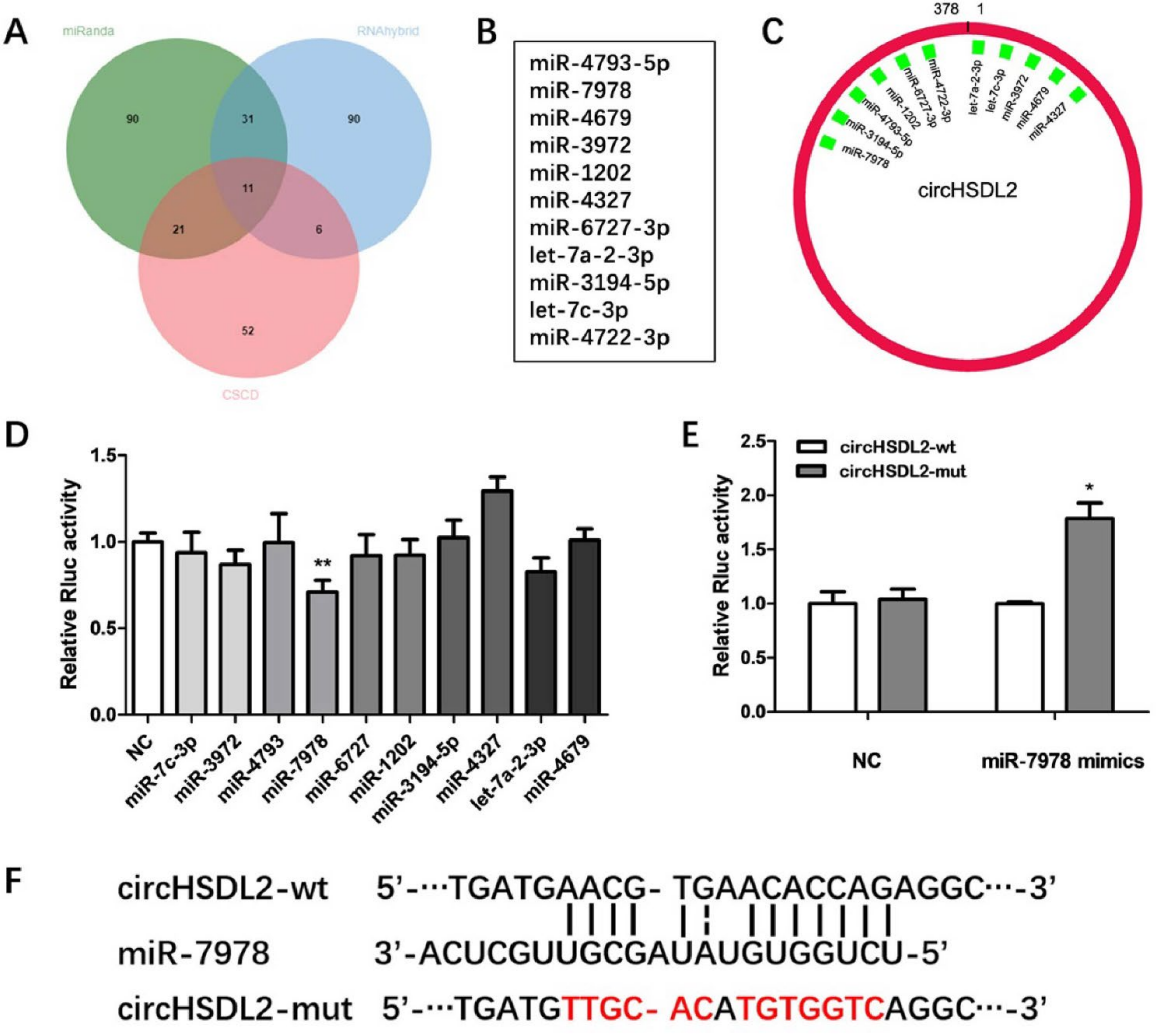
CircHSDL2 as a sponge for miR-7978 in BCa cells. **(A)** 11 predicted potential target miRNAs from three databases: RNAhybrid, miRanda, and CSCD. (**B**-**C**) 11 predicted miRNAs and their predicted binding sites with circHSDL2. **(D)** Relative Rluc activity of these candidate miRNA mimics co-transfected with wild-type circHSDL2 vectors. **(E)** Relative Rluc activity of miR-7978 mimics co-transfected with circHSDL2-wt or circHSDL2-mut vectors. **(F)** Schematic representations of circHSDL2-wt and circHSDL2-mut luciferase reporter vectors, along with the binding site of miR-7978 on circHSDL2 predicted by miRanda

Since the mechanism underlying the biological functions of circRNAs is related to subcellular localization and circHSDL2 is present in cytoplasm, we hypothesize that circHSDL2 acts as a miRNA sponge in BCa development. Then 11 potential target miRNAs of circHSDL2 were predicted by cross-analyzing RNAhybrid, miRanda and CSCD databases (Fig. 3A, B). The predicted potential binding sites were shown in Fig. 3C. Dual-luciferase reporter test was done to screen the potential miRNAs harbored by circHSDL2. The dual-luciferase reporter vectors with full-length wild circHSDL2 were constructed and co-transfected with candidate miRNA mimics or miR-NC. Luciferase activity significantly dropped in 293T cells co-transfected with miR-7978 mimics (Fig. 3D). Thus, circHSDL2 may bind to miR-7978. To verify whether miR-7978 can directly target circHSDL2, we constructed dual-luciferase reporter vectors carrying the sequence with mutant miR-7978 binding sites (Fig. 3F). After co-transfection of miR-7978 mimics and circHSDL2-wt or circHSDL2-mut, the Rluc activity remarkably increased in the circHSDL2-mut group (Fig. 3E). In all, these results imply circHSDL2 may serve as a sponge for miR-7978.

### MiR-7978 inhibits proliferation, migration and invasion in BCa cells

**Fig. 4:**
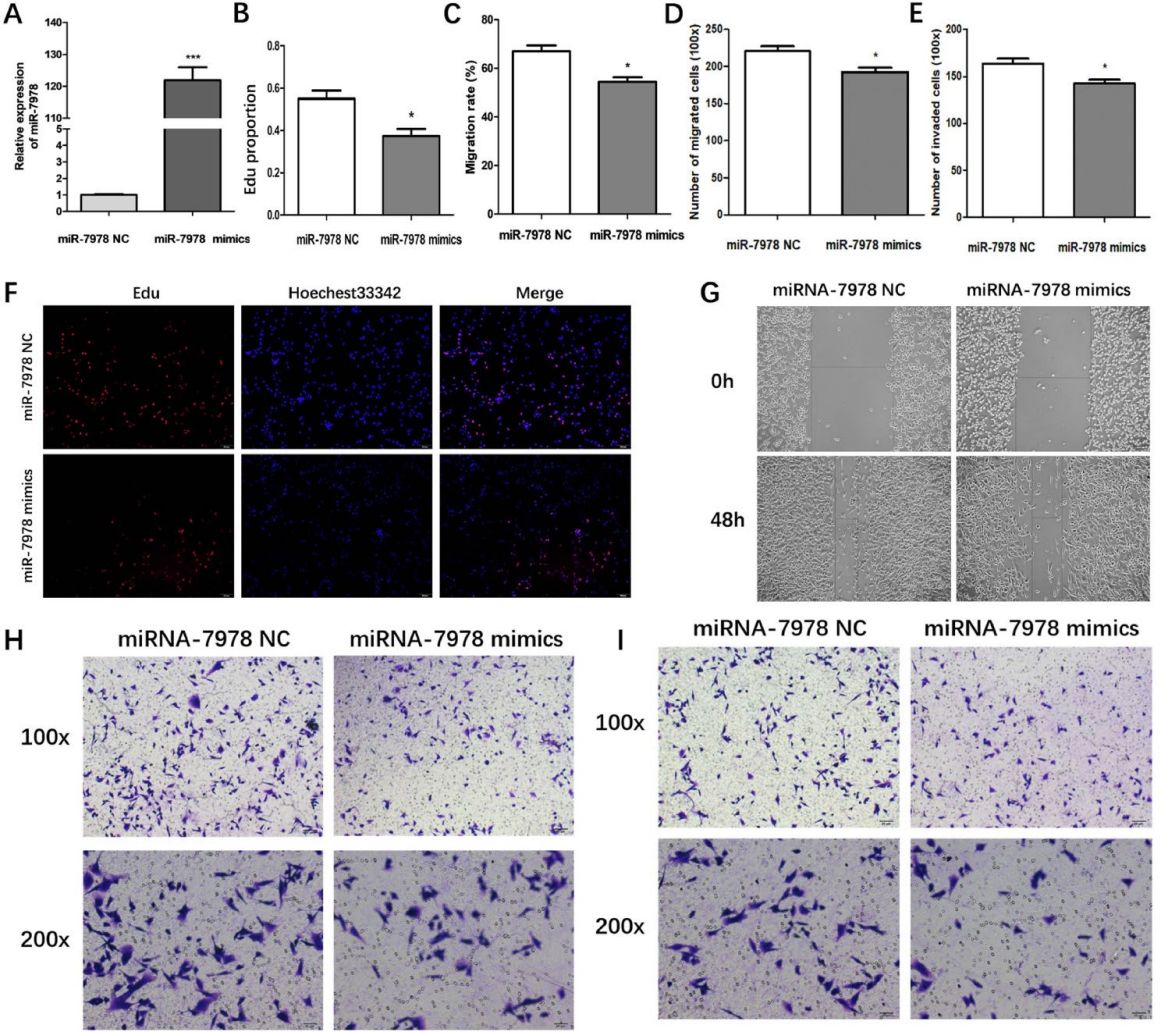
MiR-7978 restricts proliferation, migration and invasion in BCa cells. **(A)** Relative expression of miR-7978 in MDA-MB-231 cells transfected with miR-7978 mimics or negative control. (**B**, **F**) EdU assay for proliferation and (**C**, **G**) wound healing assays for migration of breast cancer cells transfected with miR-7978 mimics or negative control. (**D**, **E**, **H**, and **I**) Impact of miR-7978 mimics or negative control transfection on the migration and invasion of breast cancer cells in transwell and matrigel assays. Data were presented as mean ± SD from at least three independent experiments, *p < 0.05, **P < 0.01, ***P < 0.001

To further explore the function of miR-7978, we conducted miR-7978 overexpression in MDA-MB-231 cells and tested the proliferating, migrating and invading abilities. qRT-PCR uncovered the overexpressing effectiveness of miR-7978 (Fig. 4A). Edu assays demonstrated that miR-7978 overexpression reduced the percent of EdU-positive cells in BCa cells (Fig. 4B, F). Wound healing and transwell assays revealed that miR-7978 overexpression lowered the migrating and invading abilities of BCa cells (Fig. 4C-E, G-I). The results above suggest that miR-7978 may serve as an antineoplastic factor to inhibit the proliferation, migration and invasion in BCa cells.

### ZNF704 and MST2 are downstream effector proteins of circHSDL2 and miR-7978

**Fig. 5:**
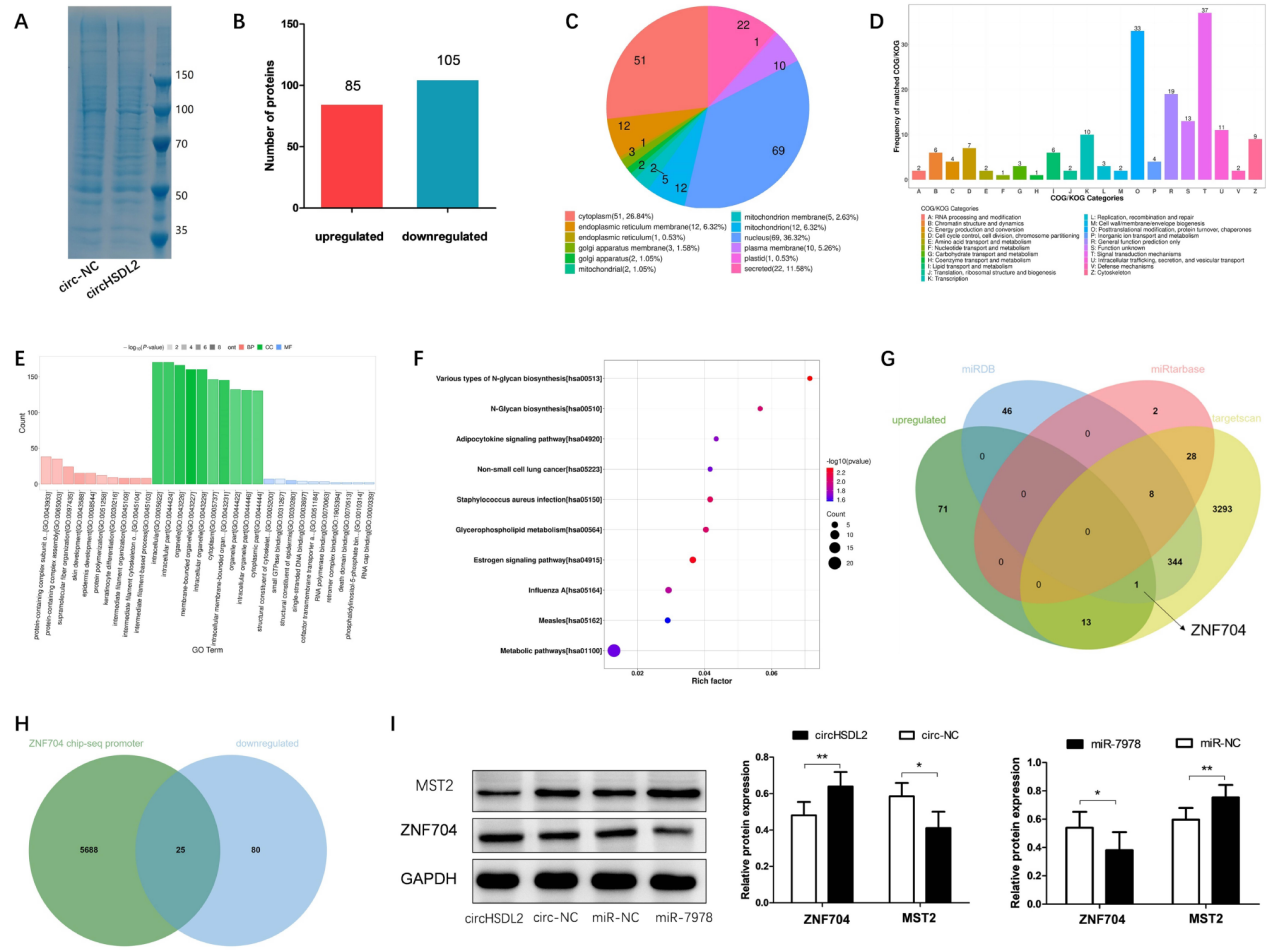
Proteomic analysis and Western blotting validation. **(A)** SDS-PAGE combined with Coomassie brilliant blue staining of protein samples from circHSDL2 overexpression group and negative control group. **(B)** Bar chart on distribution of differentially expressed proteins (DEPs) in the two groups (criteria for selection: fold change ≤ 0.5 or ≥ 2). **(C)** Pie chart on subcellular positioning analysis of DEPs. **(D)** Histogram on COG analysis of DEPs. **(E)** Categorical histogram on GO enrichment analysis of DEPs. **(F)** Bubble chart on KEGG metabolic pathway enrichment analysis of DEPs. **(G)** Venn diagram on intersection of predicted target genes of miR-7978 in the miRDB, miRtarbase, and targetscan databases and the upregulated proteins in the proteomics data. **(H)** Intersection of potential downstream target proteins of ZNF704 obtained from ChIP-seq and proteins downregulated in the proteomics data. **(I)** Expression level changes of ZNF704 and MST2 after circHSDL2 and miR-7978 overexpression. Data were expressed as mean ± SD from at least three independent assays, *p < 0.05, **P < 0.01

To further investigate the downstream target genes of circHSDL2 through miR-7978 sequestration, we conducted proteomics analysis in breast cancer cells overexpressing circHSDL2 and a negative control. SDS-PAGE combined with Coomassie brilliant blue staining clearly revealed distinct protein bands in both groups, with an even distribution of protein molecular weights (Fig. 5A). We identified differentially expressed proteins (DEPs) based on FC ≥ 2 or ≤ 0.5, resulting in totally 85 upregulated and 105 downregulated proteins (Fig. 5B). Subcellular localization analysis of these DEPs was visualized in a pie chart, showing that most of these proteins were localized in the cytoplasm and nucleus (Fig. 5C). To gain deep insights into the roles of these DEPs, we performed Cluster of Orthologous Groups of proteins (COG) (Fig. 5D). Among these proteins, various clusters were observed in categories related to ‘posttranslation modification, protein turnover, chaperones’ and ‘signal transduction mechanism’. We then mapped the differentially expressed genes (DEGs) to the Human Gene Ontology database and analyzed functional enrichment using GO (http://www.geneontology.org/) (Fig. 5E). For a more comprehensive understanding, we utilized the Kyoto Encyclopedia of Genes and Genomes (KEGG) Pathway database (www.kegg.jp/kegg/pathway.html) to detect the metabolic pathways significantly enriched with DEPs (Fig. 5F).

In our quest to identify common downstream target genes of both circHSDL2 and miR-7978, we forecast the target genes of miR-7978 using miRDB, miRtarbase, and targetscan databases. We then intersected these predictions with the upregulated DEGs identified earlier (Fig. 5G). Notably, one protein, ZNF704, was found to be a common target of miR-7978 as predicted by miRDB and targetscan. Further review of the literature shows that ZNF704 is a transcriptional repressor potentially implicated in tumor development through pathways like Hippo signaling, circadian rhythm, and MAPK signaling [16].

To validate our findings, the results from chromatin immunoprecipitation- based deep sequencing (ChIP-seq) of ZNF704 from the literature were overlapped with the downregulated genes from our proteomics analysis (Fig. 5H). Totally 25 common downstream targets shared by circHSDL2 and ZNF704 were found (Table S2). Interestingly, MST2, a crucial part of Hippo signaling, was among them. We hypothesize that ZNF704 and MST2 may serve as downstream targets of circHSDL2 and miR-7978. To confirm this hypothesis, we conducted Western blotting, which revealed that circHSDL2 overexpression significantly upregulated ZNF704 and downregulated MST2, while miR-7978 overexpression had the opposite effect (Fig. 5I). Hence, our results suggest that circHSDL2 modulates ZNF704 expression by sequestering miR-7978 and may facilitate the malignant development of breast cancer through the Hippo signaling pathway.

### CircHSDL2 promotes breast cancer progression through circHSDL2/miR-7978/ZNF704 axis and dysregulating Hippo signaling pathway

**Fig. 6:**
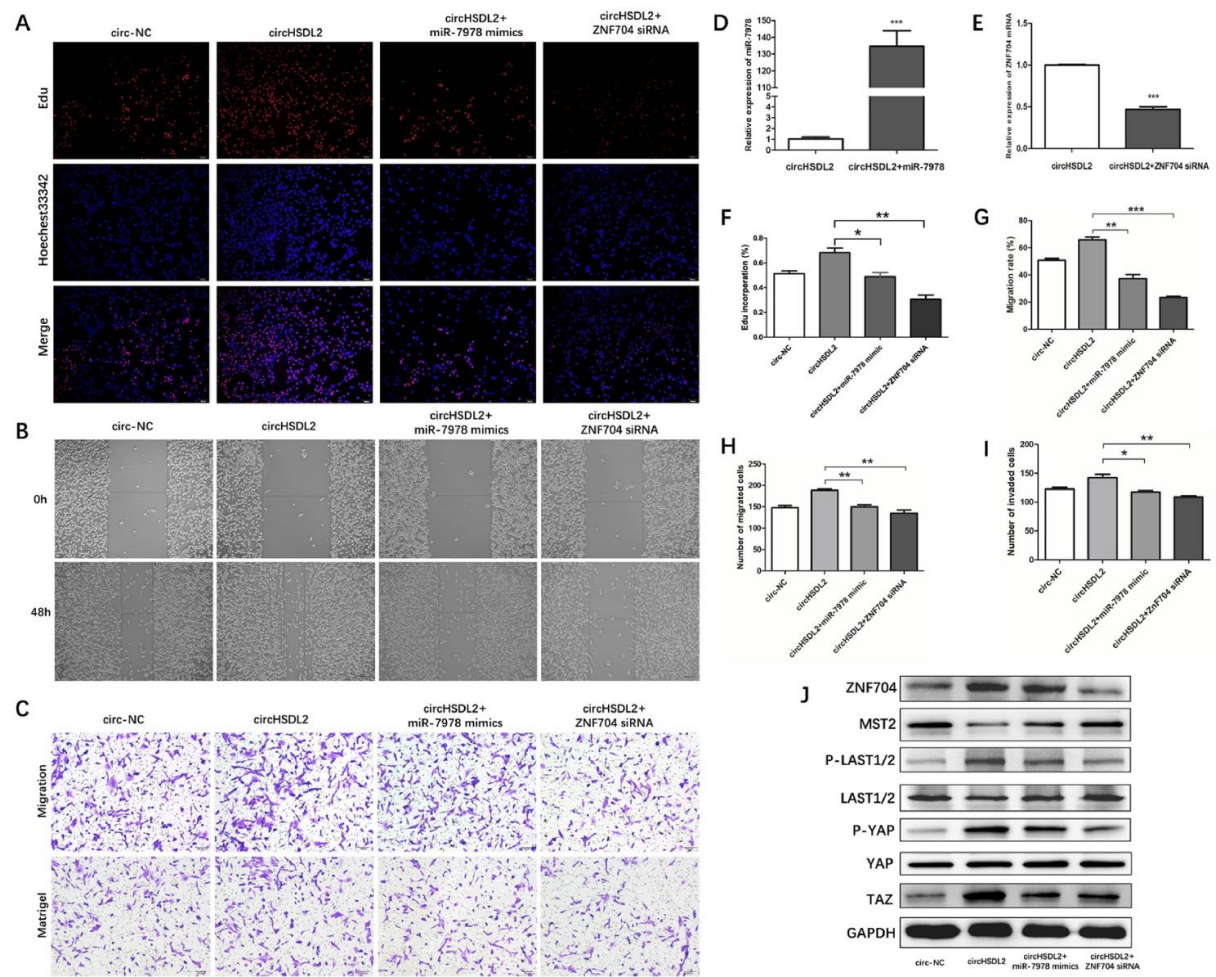
CircHSDL2 functions through the circHSDL2/miR-7978/ZNF704 axis and dysregulating Hippo signaling pathway. (**A**, **F**) EdU assay for proliferation, (**B**, **G**) wound healing assay for migration, and (**C**, **H**, and **I**) transwell migration and matrigel invasion assays for migration and invasion abilities of breast cancer cells transfected with circHSDL2, circHSDL2 + miR-7978 mimics, circHSDL2 + ZNF704 siRNA or negative control. Relative expressions in MDA-MB-231 cells **(D)** of miR-7978 after transfection with circHSDL2 and circHSDL2 + miR-7978 mimics, and **(E)** of ZNF704 mRNA after transfection with circHSDL2 and circHSDL2 + ZNF704 siRNA. **(J)** Western blotting of ZNF704 and relevant proteins in the hippo signaling pathway in the four groups. Data were shown as mean ± SD from at least three independent assays, *p < 0.05, **P < 0.01

To further validate the mechanism of circHSDL2, we conducted a series of rescue experiments to elucidate the underlying mechanism by which the circHSDL2/miR-7978/ZNF704 axis promotes breast cancer progression. EdU experiments showed that circHSDL2 overexpression remarkably augmented the proliferation capability of MDA-MB-231 cells. Notably, this enhancement was abrogated by the introduction of miR-7978 mimics and ZNF704 siRNA (Fig. 6A and F). The scratch assays uncovered that circHSDL2 overexpression substantially heightened the migratory capacity of MDA-MB-231 cells. This effect was also counteracted by miR-7978 mimics and ZNF704 siRNA (Fig. 6B and G). Furthermore, transwell migration and invasion assays yielded consistent results with the findings in the scratch assays, further affirming the impact of circHSDL2 on cell migration and invasion (Fig. 6C and H, and 6I). Figure 6D and E illustrates the effects of miR-7978 mimics and ZNF704 siRNA on the mRNA expression levels of miR-7978 and ZNF704, respectively. Given that MST2 is pivotal in the Hippo pathway, which is known to participate in the growth of diverse tumors, this study proves the influence of circHSDL2 and miR-7978 on MST2 expression levels. Consequently, we employed Western blotting to assess protein levels of the Hippo pathway. Results indicate that circHSDL2 overexpression leads to a reduction in MST2 expression levels, while elevating the levels of ZNF704, P-LAST1/2, P-YAP, and TAZ. Importantly, these changes induced by circHSDL2 overexpression can be partially reversed by miR-7978 mimics and ZNF704 siRNA (Fig. 6J). Collectively, these findings suggest that circHSDL2 may modulate ZNF704 expression through miR-7978 sequestration, and subsequently impacts the Hippo signaling pathway.

#### CircHSDL2 promotes breast cancer progression in vivo

**Fig. 7:**
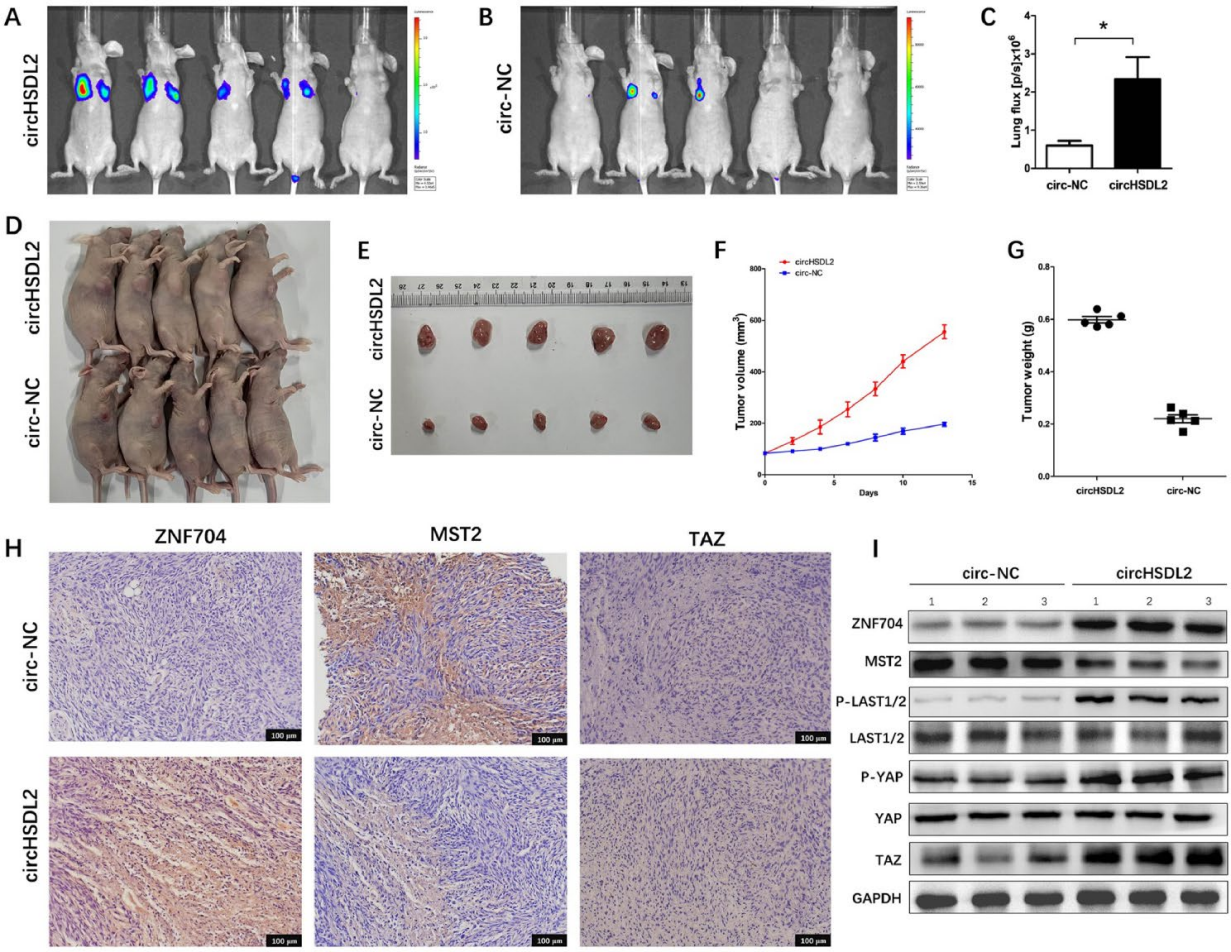
(**A**-**C**) A lung metastasis model built by tail vein injection of breast cancer cells. In vivo imaging of lung metastatic tumors in mice using fluorescence imaging. (**D**-**G**) MDA-MB-231 cells (circHSDL2 group and circ-NC group) were subcutaneously implanted into female nude mice. The tumor volume and weight in the circHSDL2 group were significantly larger than those in the circ-NC group. **(H)** Representative Datas of immunohistochemistry for ZNF704, MST2 and TAZ in the two groups. **(I)** Proteins in the tumor tissues of both groups detected by Western blotting for ZNF704 and the relevant proteins in the Hippo pathway. Data were reported as mean ± SD from at least three tests *p < 0.05, **P < 0.01

To probe into the role of circHSDL2 in the malignant progression of breast cancer in vivo, we conducted xenograft tumor experiments using lentivirus-infected MDA-MB-231 cells. Initially, we built a lung metastasis model by injecting caudal vein of female nude mice with circHSDL2-overexpressing breast cancer cells and the control group consisting of circ-NC breast cancer cells. We monitored lung metastasis in both groups through in vivo imaging (Fig. 7A-C). The data clearly indicate that circHSDL2 overexpression significantly promotes lung metastasis in breast cancer. We then subcutaneously implanted both groups of cells into female nude mice and monitored tumor growth and volume. Compared to the circ-NC group, tumors in the circHSDL2 group grew faster and were larger in size (Fig. 7D-G). We also did immunohistochemistry (IHC) to evaluate the expressions of ZNF704, MST2, and TAZ. The ZNF704 and TAZ expressions increased, while the MST2 expression decreased in the circHSDL2 group (Fig. 7H). In addition, we extracted proteins from xenograft tumors and conducted Western blotting and IHC to detect the expressions of ZNF704, MST2, TAZ, YAP, P-YAP, LATS1/2, and P-LATS1/2 (Fig. 7I). Results confirmed that in the circHSDL2 overexpression group, the expressions of ZNF704, P-YAP, P-LATS1/2, and TAZ increased, while MST2 expression decreased. Collectively, these findings demonstrate that circHSDL2 promotes the proliferation and metastasis of breast cancer cells in vivo. Given all the in vivo and in vitro experimental results, it is evident that circHSDL2 dysregulates the Hippo signaling pathway through the circHSDL2/miR-7978/ZNF704 axis, and ultimately drives breast cancer progression.

## Discussion

With the advancement of high-throughput sequencing tools, more and more circRNAs are being discovered [17]. As a novel class of gene regulatory factors, circRNAs play roles in transcriptionally or post-transcriptionally regulating downstream factors [18]. Mounting evidence suggests that circRNAs may be expressed differently in tumor tissues and participate in breast cancer progression in various ways [19–22]. However, their functions are not yet fully understood. In our study, analysis of circRNA expression profiles in tissues and exosomes uncovers a significant upregulation of circHSDL2. Subsequently, we conducted functional experiments to probe into the action of circHSDL2 in malignant development of breast cancer cells.

First, functional gain-of-function experiments were done in breast cancer cells (MDA-MB-231 cells) to study the functional role of circHSDL2. Results indicated that circHSDL2 overexpression facilitated the division, movement and invasion of breast cancer cells. The experimental data suggest that circHSDL2 indeed plays a functional regulatory role in breast cancer pathogenesis. The next step was to elucidate the molecular mechanism underlying the functional role of circHSDL2. For this purpose, we used bioinformatic analysis to forecast the potential targets of circHSDL2 and ultimately verified via dual-luciferase reporter assays that miR-7978 is one biological target of circHSDL2. MiRNAs are a class of non-coding RNAs of 22–25 nucleotides in length that function by targeting the 3’-UTR of mRNA to restrict gene expression. Currently, there is limited report on the functions and role of miR-7978 in breast cancer. Our study found that miR-7978 overexpression significantly restricted the proliferation, motion and invasion of breast cancer cells. We identified the downstream target gene ZNF704 shared between circHSDL2 and miR-7978 through proteomics and bioinformatics analysis and further confirmed it through Western blotting. ZNF704 is a transcriptional repressor that may be involved in tumor development through various signaling pathways, including the Hippo pathway. By analyzing the potential protein interactions reported before and the downstream proteins of circHSDL2 in proteomics, we found that MST2 may be a downstream target of circHSDL2/miR-7978/ZNF704. MST2 is a core component of the Hippo pathway, which is involved in the growth of various cancers. On this basis, we hypothesize that circHSDL2 may regulate ZNF704 expression by sequestering miR-7978 and may participate in the malignant progression of breast cancer through the Hippo pathway.

To validate these hypotheses, we used a cotransfection system with circHSDL2 and miR-7978 mimics and found that interference with miR-7978 mimic attenuated the impact of circHSDL2 upregulation on cell division, movement and invasion, indicating that circHSDL2 may affect breast cancer progression by modulating miR-7978. We also used Western blotting to verify the involvement of ZNF704, MST2, and the Hippo pathway in this process. We observed that circHSDL2 overexpression led to a drop in MST2 expression and a rise in ZNF704, P-LAST1/2, P-YAP and TAZ expressions. Importantly, these changes induced by circHSDL2 overexpression can be partially reversed by miR-7978 mimic and ZNF704 siRNA. This result confirms that circHSDL2 modulates the Hippo pathway by sequestering miR-7978, and thereby participates in the malignancy of breast cancer. Further in vivo assays confirm that circHSDL2 overexpression can promote breast cancer proliferation and lung metastasis.

In conclusion, circHSDL2 can facilitate breast cancer proliferation, motion and invasion. Mechanistically, circHSDL2 acts as a competitive endogenous RNA (ceRNA) for miR-7978 to regulate ZNF704 expression and the Hippo pathway. Based on our data, circHSDL2 may be a target of breast cancer treatment, and further clinical studies involving more patients are needed to establish its clinical significance.

### Electronic supplementary material

Below is the link to the electronic supplementary material.

Supplementary Material 1

Supplementary Material 2

Supplementary Material 3

Supplementary Material 4

Supplementary Material 5

Supplementary Material 6

Supplementary Material 7

Supplementary Material 8

Supplementary Material 9

Supplementary Material 10

Supplementary Material 11

Supplementary Material 12

Supplementary Material 13

Supplementary Material 14

Supplementary Material 15

Supplementary Material 16

Supplementary Material 17

Supplementary Material 18

Supplementary Material 19

Supplementary Material 20

Supplementary Material 21

## Data Availability

No datasets were generated or analysed during the current study.
