# Supplementary material for: Circular RNA HSDL2 promotes breast cancer progression via miR-7978 ZNF704 axis and regulating hippo signaling pathway
Source: Breast Cancer Res. 2024 Jun 27;26:105. doi: 10.1186/s13058-024-01864-z (PMC11210124; doi:10.1186/s13058-024-01864-z)
Supplement: Supplementary file 1 — Supplementary Material 1 [file 13058_2024_1864_MOESM1_ESM.docx]

Table S1 Sequences of all primers and siRNAs used in this study

| **Name** | **Sequence (5'to3')** |
| --- | --- |
| miRNA-7978-forward ACACTCCAGCTGGGTCTGGTGTATAGCGT | ACACTCCAGCTGGGTCTGGTGTATAGCGT |
| miRNA-7978-reverse | CTCAACTGGTGTCGTGGAGTCGGCAATTCAGTTGAGTGAGCAAC |
| U6-forward | CTCGCTTCGGCAGCACA |
| U6-reverse | AACGCTTCACGAATTTGCGT |
| circHSDL2-forward | TCTGATGATGAACGTGAACACC |
| circHSDL2-reverse | CGGCTTGCACCTGTGATAAA |
| ZNF704 siRNA | CAAUGGUACUAACCAGCUUGU |
| siRNA control | UUCUCCGAACGUGUCACGU |
