# Supplementary figures and images for: Circular RNA HSDL2 promotes breast cancer progression via miR-7978 ZNF704 axis and regulating hippo signaling pathway
Source: Breast Cancer Res. 2024 Jun 27;26:105. doi: 10.1186/s13058-024-01864-z (PMC11210124; doi:10.1186/s13058-024-01864-z)

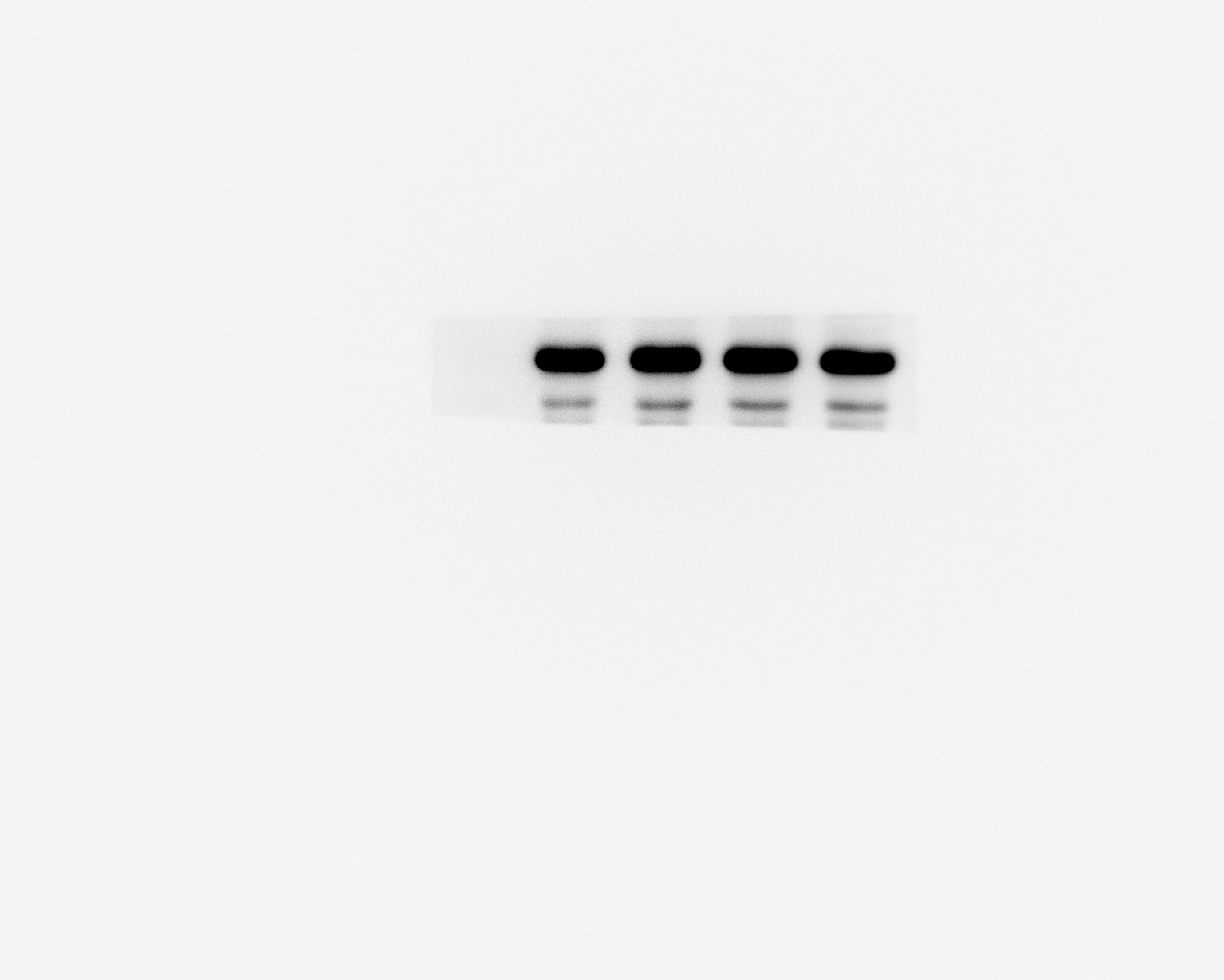

Supplement: Supplementary file 3 — Supplementary Material 3 [file 13058_2024_1864_MOESM3_ESM.tif]

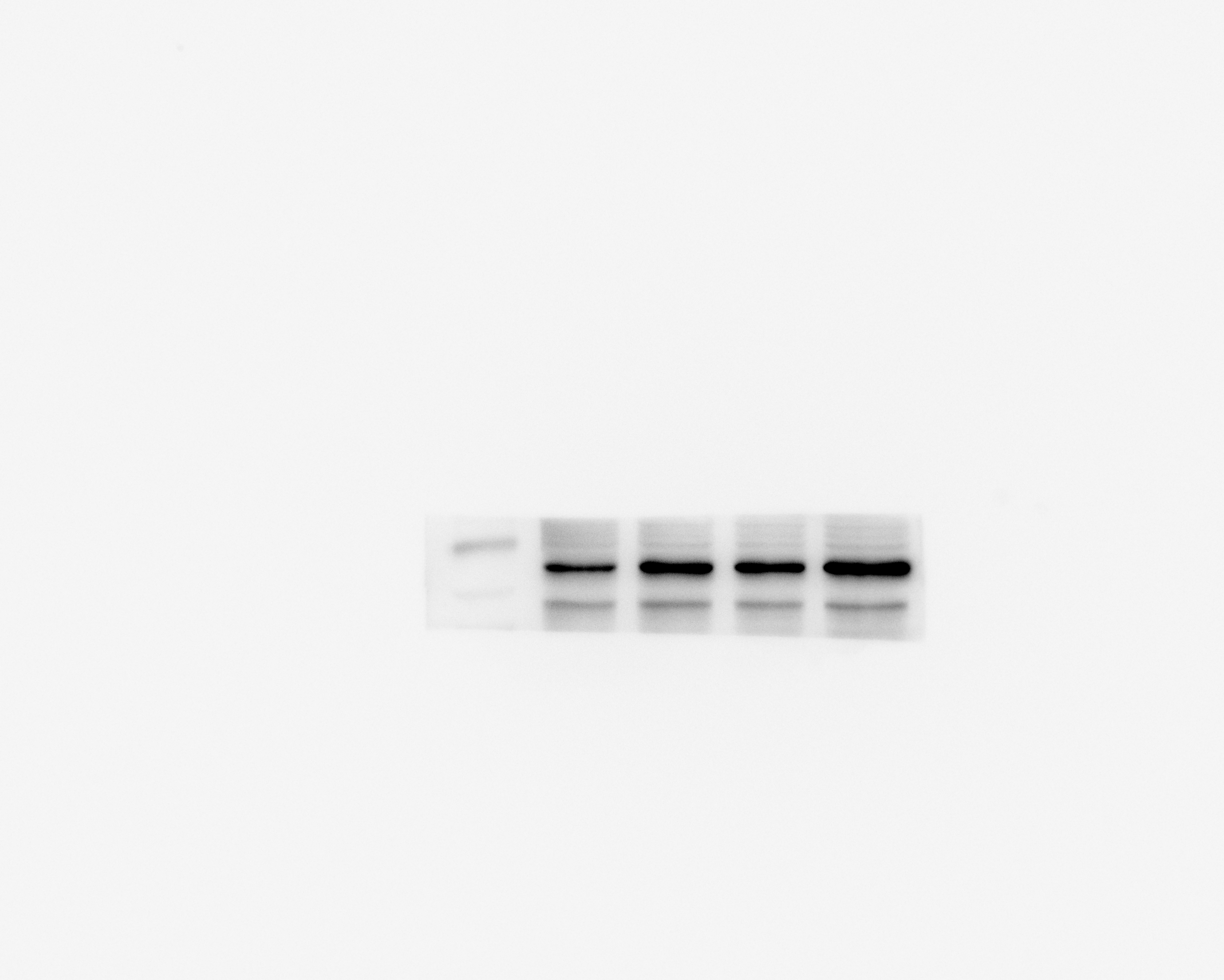

Supplement: Supplementary file 4 — Supplementary Material 4 [file 13058_2024_1864_MOESM4_ESM.tif]

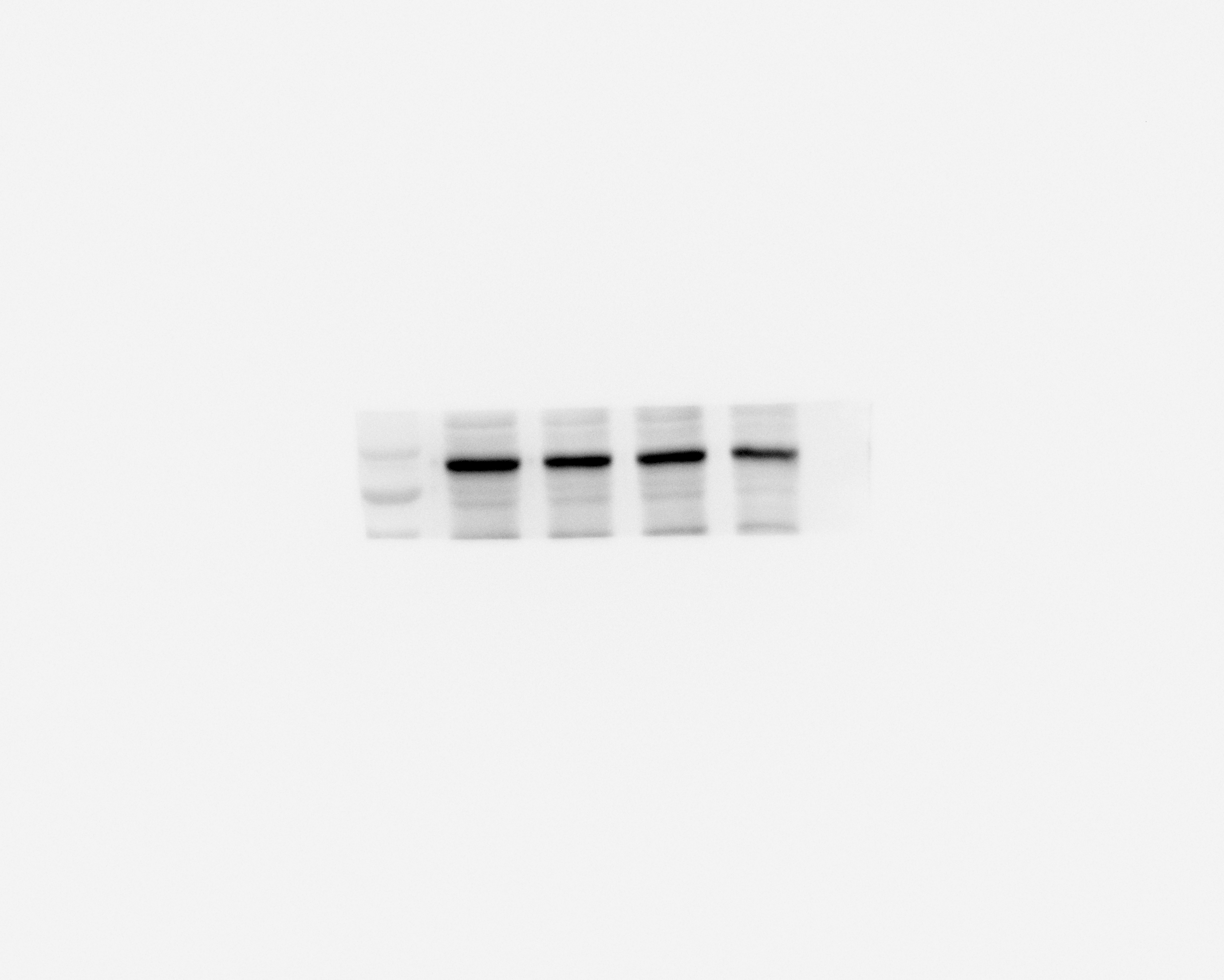

Supplement: Supplementary file 5 — Supplementary Material 5 [file 13058_2024_1864_MOESM5_ESM.tif]

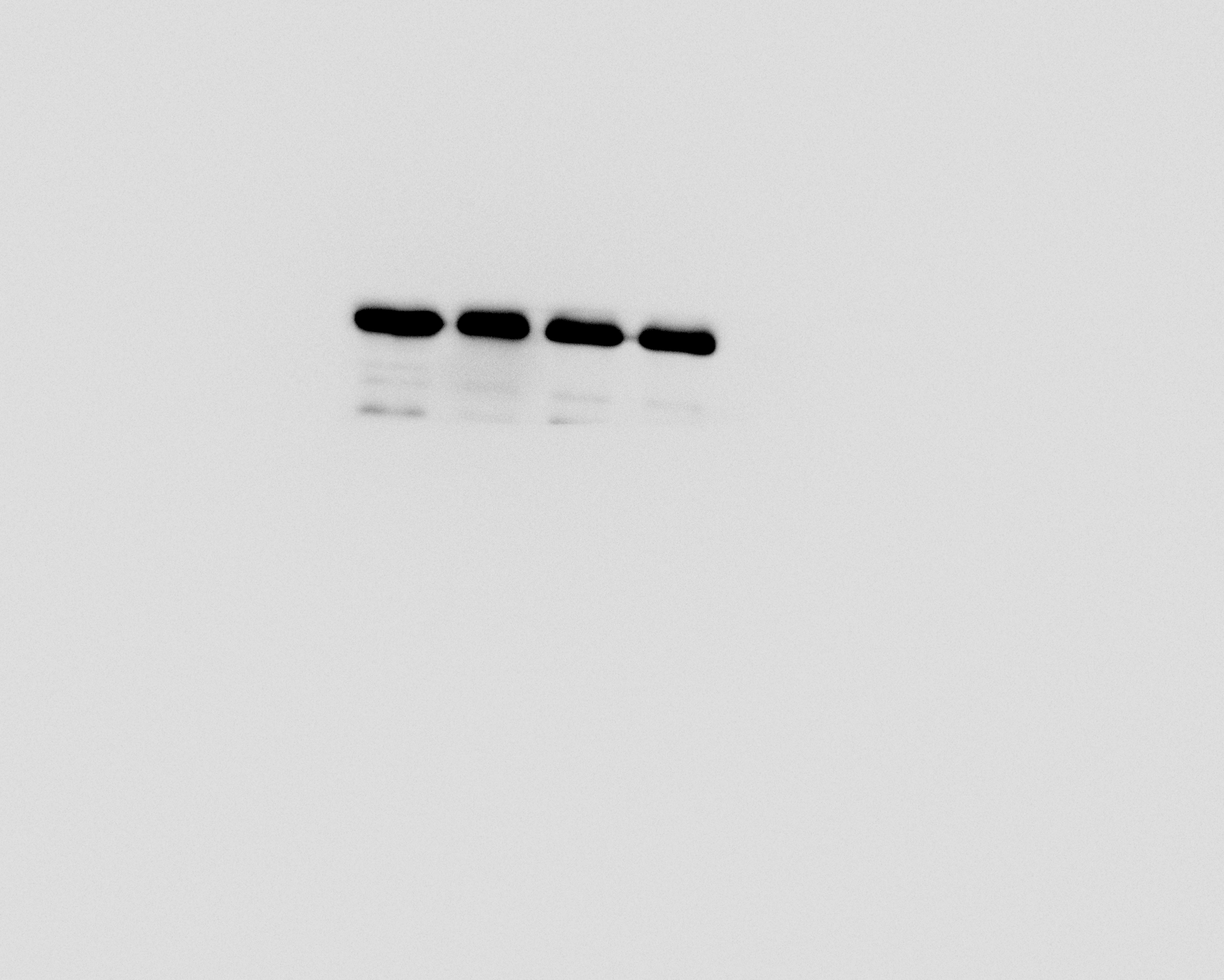

Supplement: Supplementary file 6 — Supplementary Material 6 [file 13058_2024_1864_MOESM6_ESM.tif]

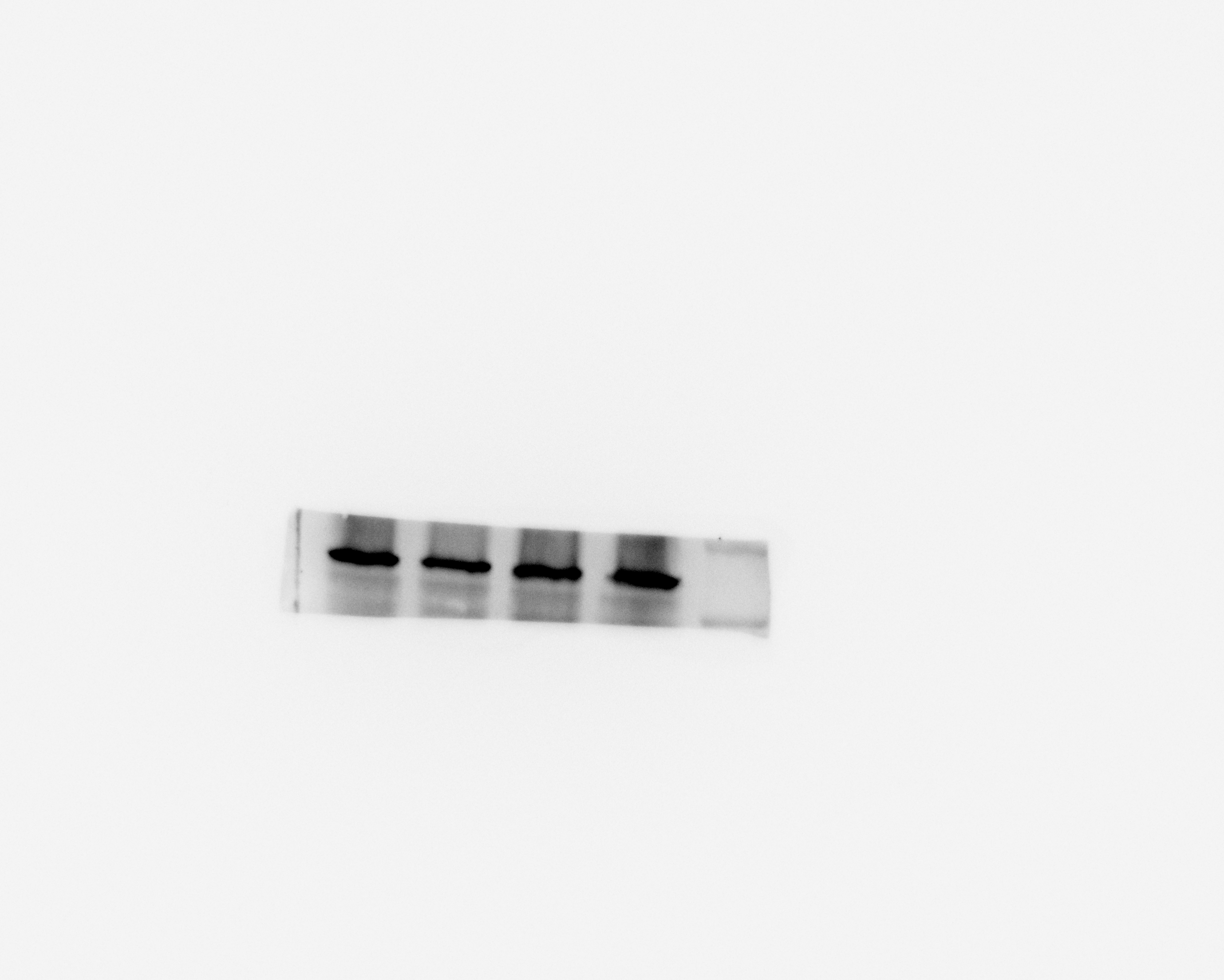

Supplement: Supplementary file 7 — Supplementary Material 7 [file 13058_2024_1864_MOESM7_ESM.tif]

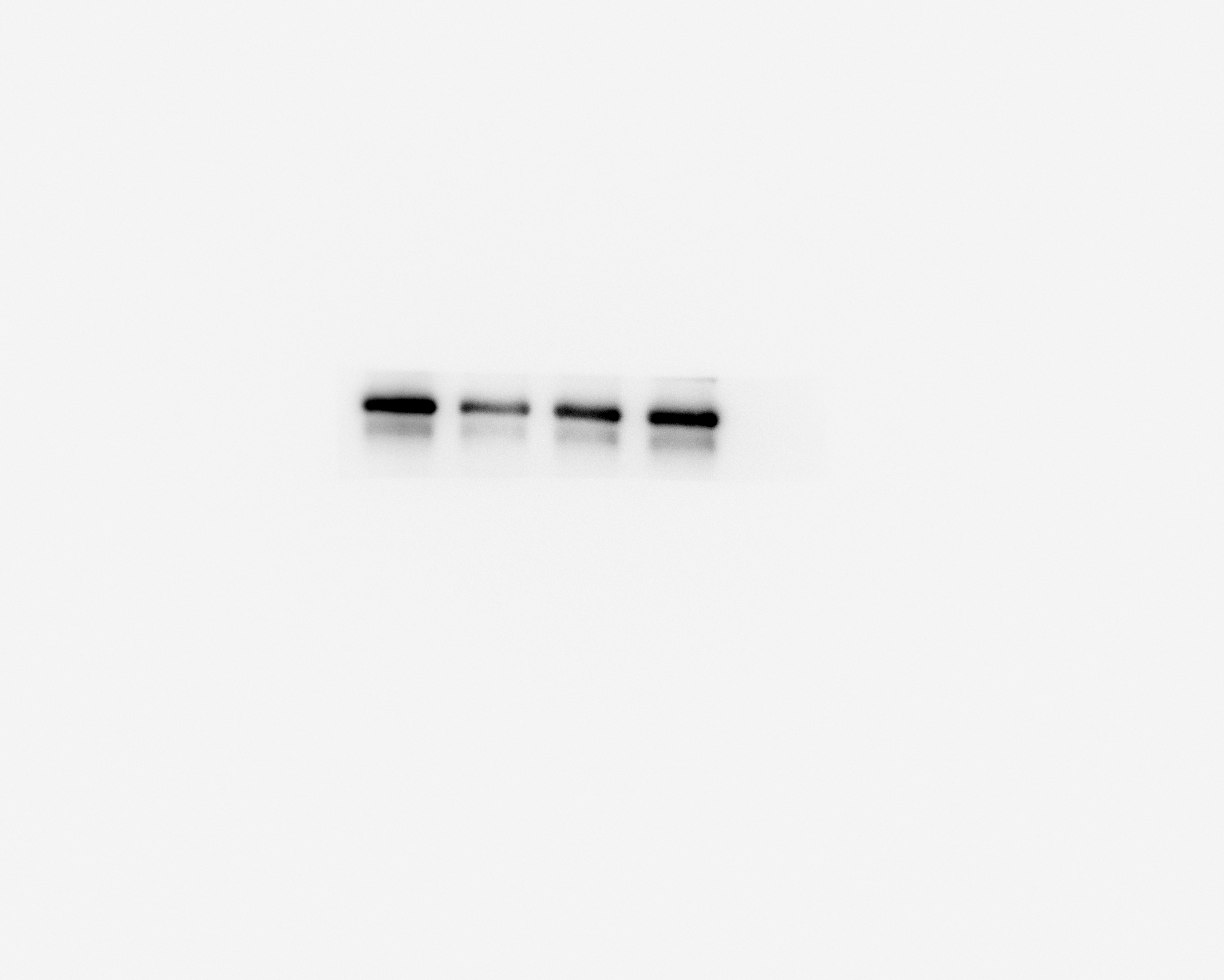

Supplement: Supplementary file 8 — Supplementary Material 8 [file 13058_2024_1864_MOESM8_ESM.tif]

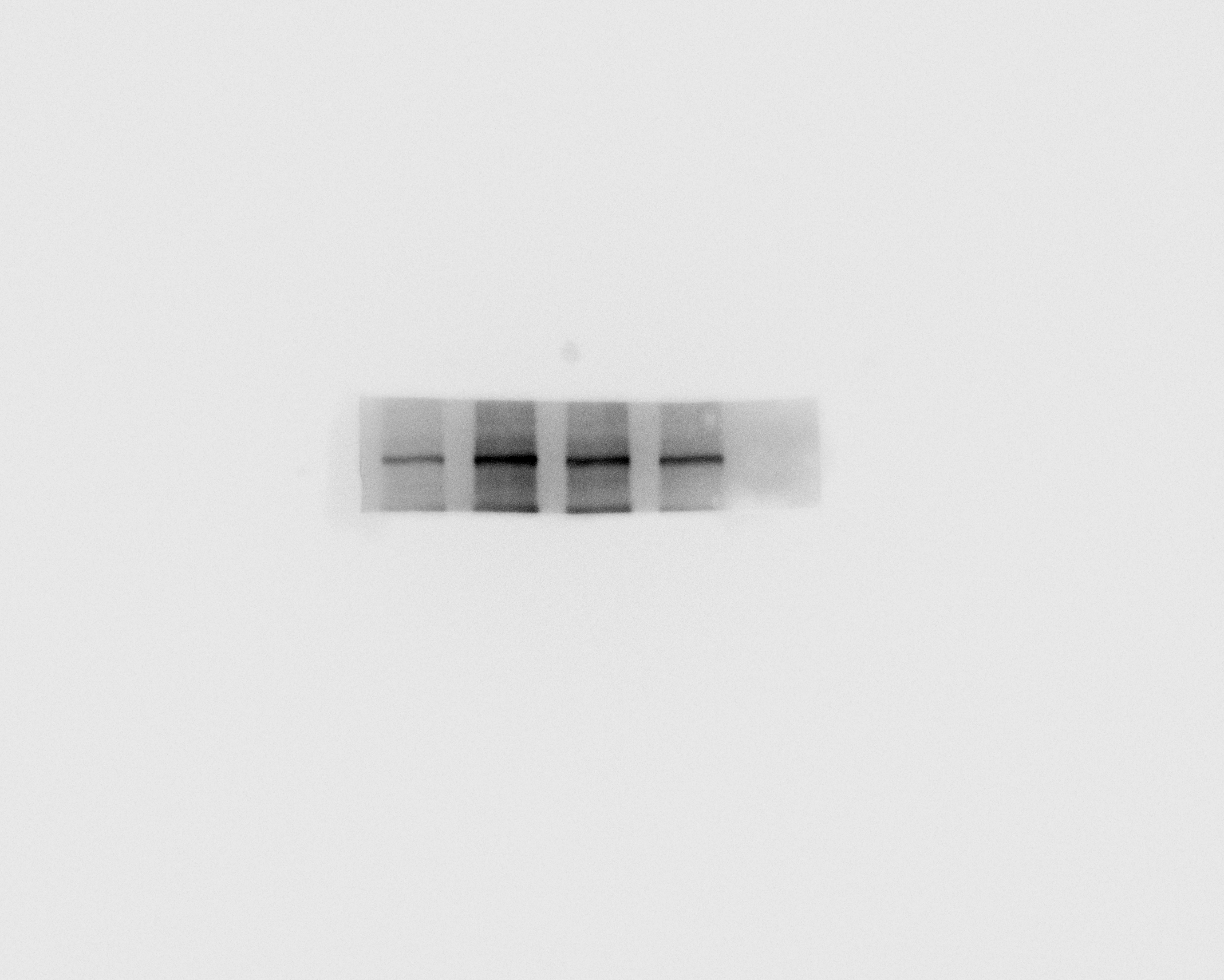

Supplement: Supplementary file 9 — Supplementary Material 9 [file 13058_2024_1864_MOESM9_ESM.tif]

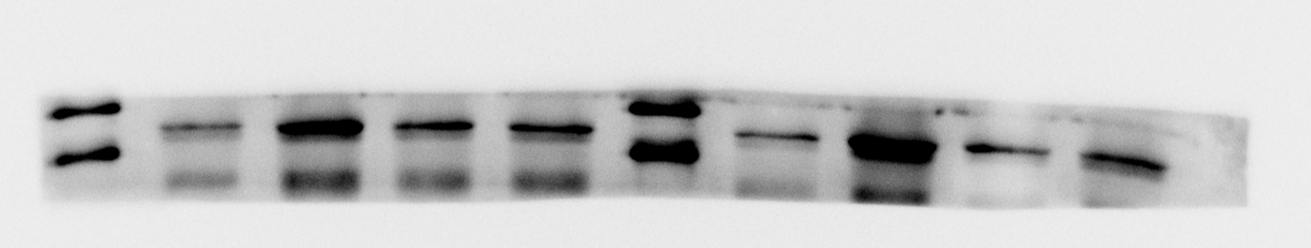

Supplement: Supplementary file 10 — Supplementary Material 10 [file 13058_2024_1864_MOESM10_ESM.tif]

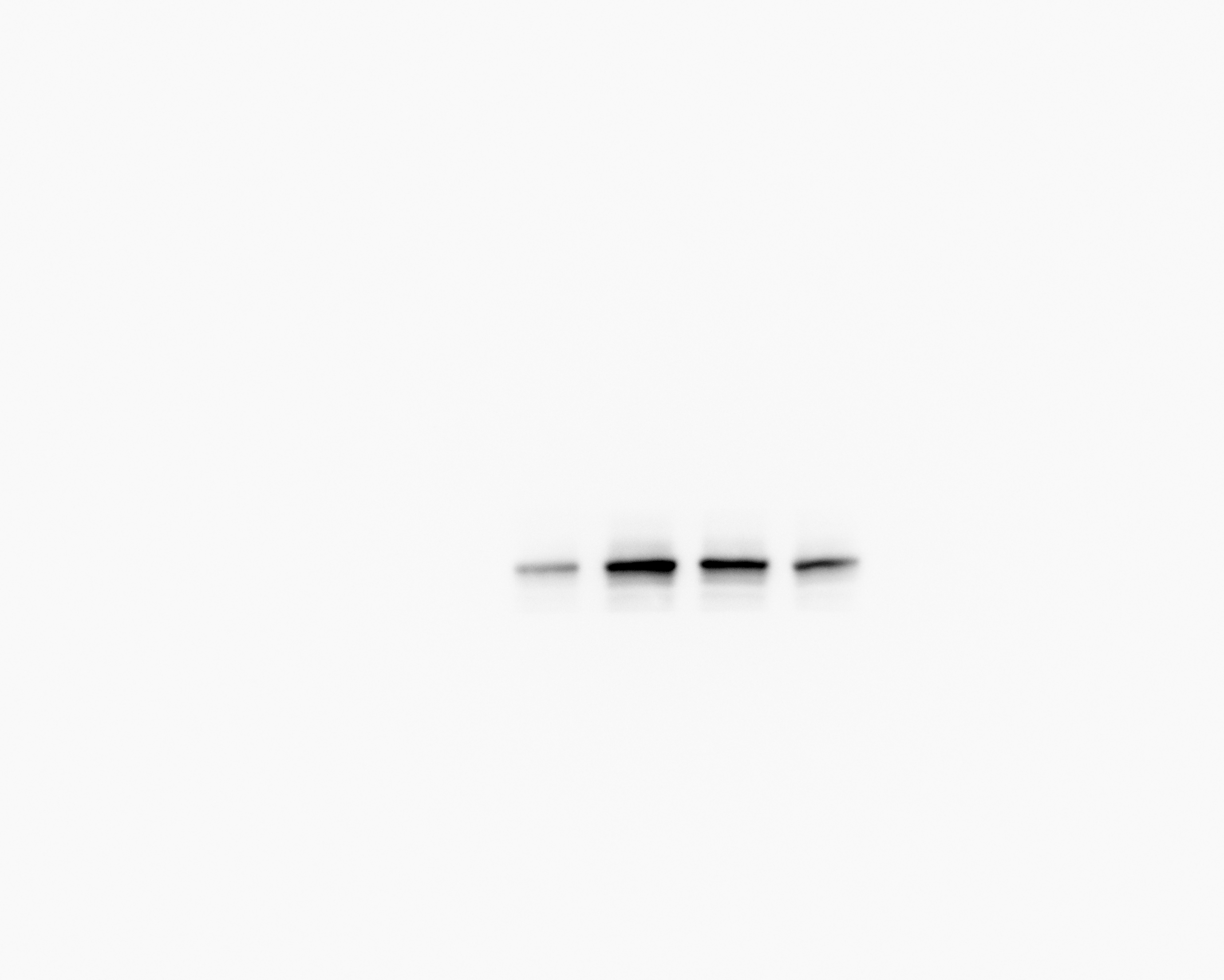

Supplement: Supplementary file 11 — Supplementary Material 11 [file 13058_2024_1864_MOESM11_ESM.tif]

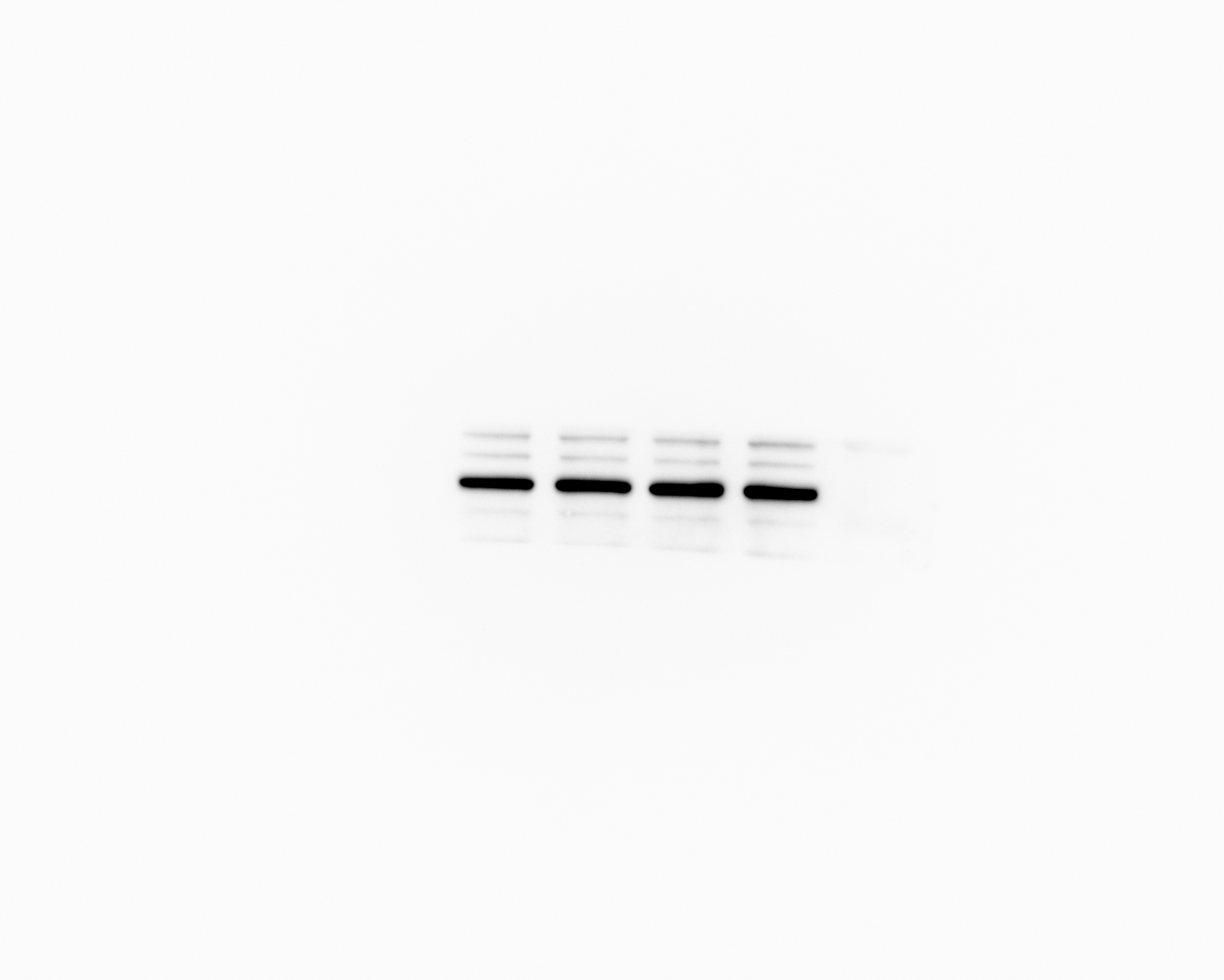

Supplement: Supplementary file 12 — Supplementary Material 12 [file 13058_2024_1864_MOESM12_ESM.tif]

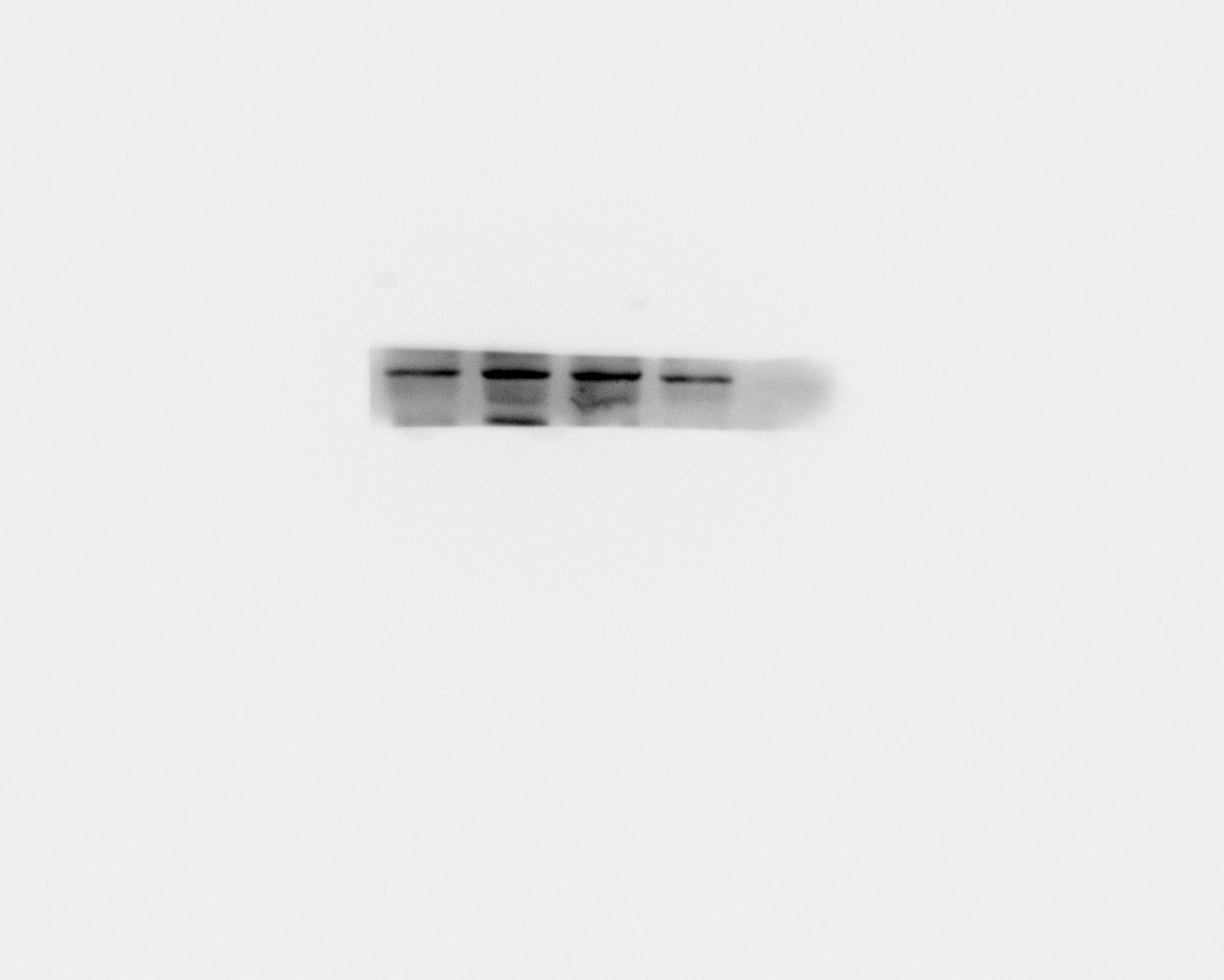

Supplement: Supplementary file 13 — Supplementary Material 13 [file 13058_2024_1864_MOESM13_ESM.tif]

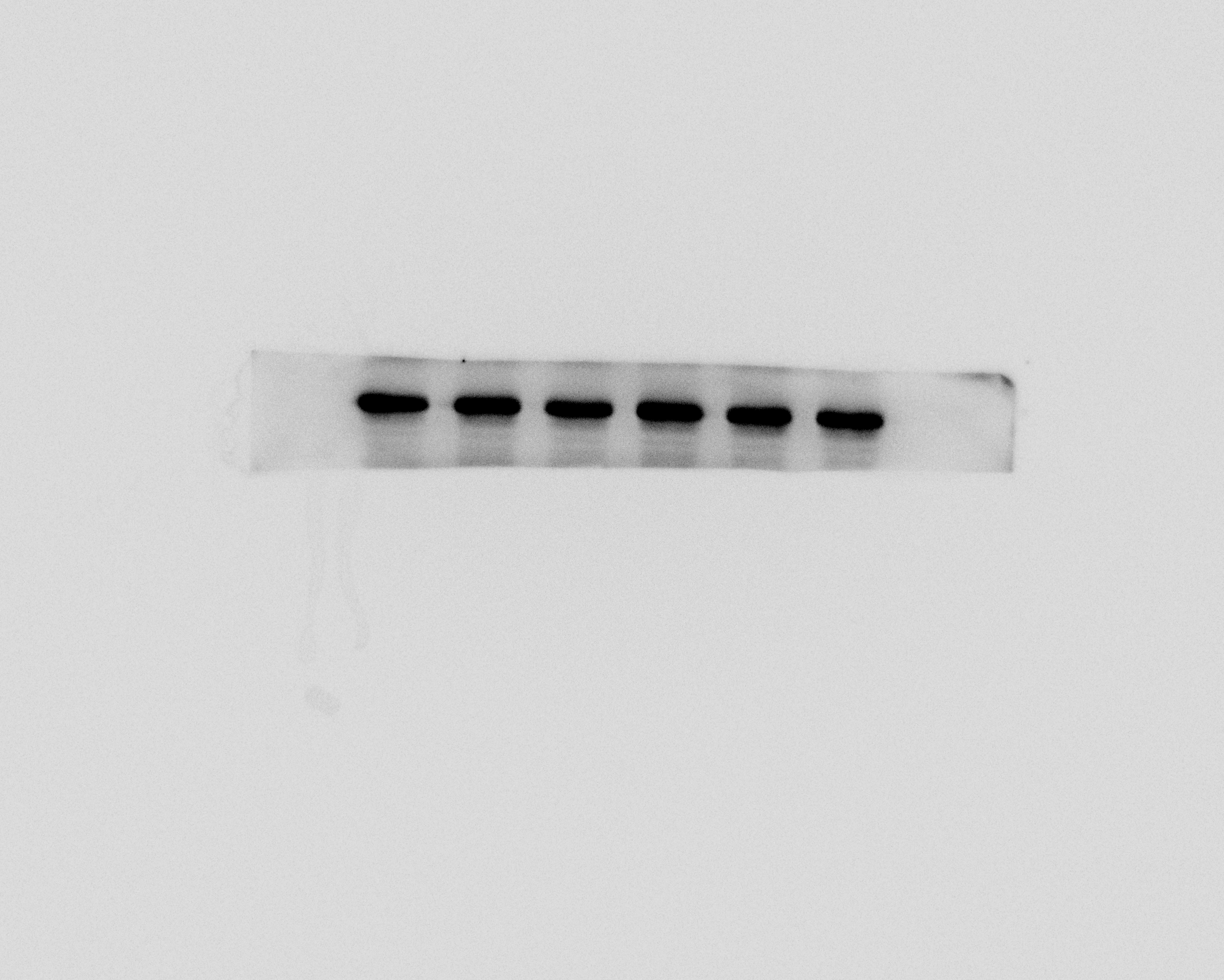

Supplement: Supplementary file 14 — Supplementary Material 14 [file 13058_2024_1864_MOESM14_ESM.tif]

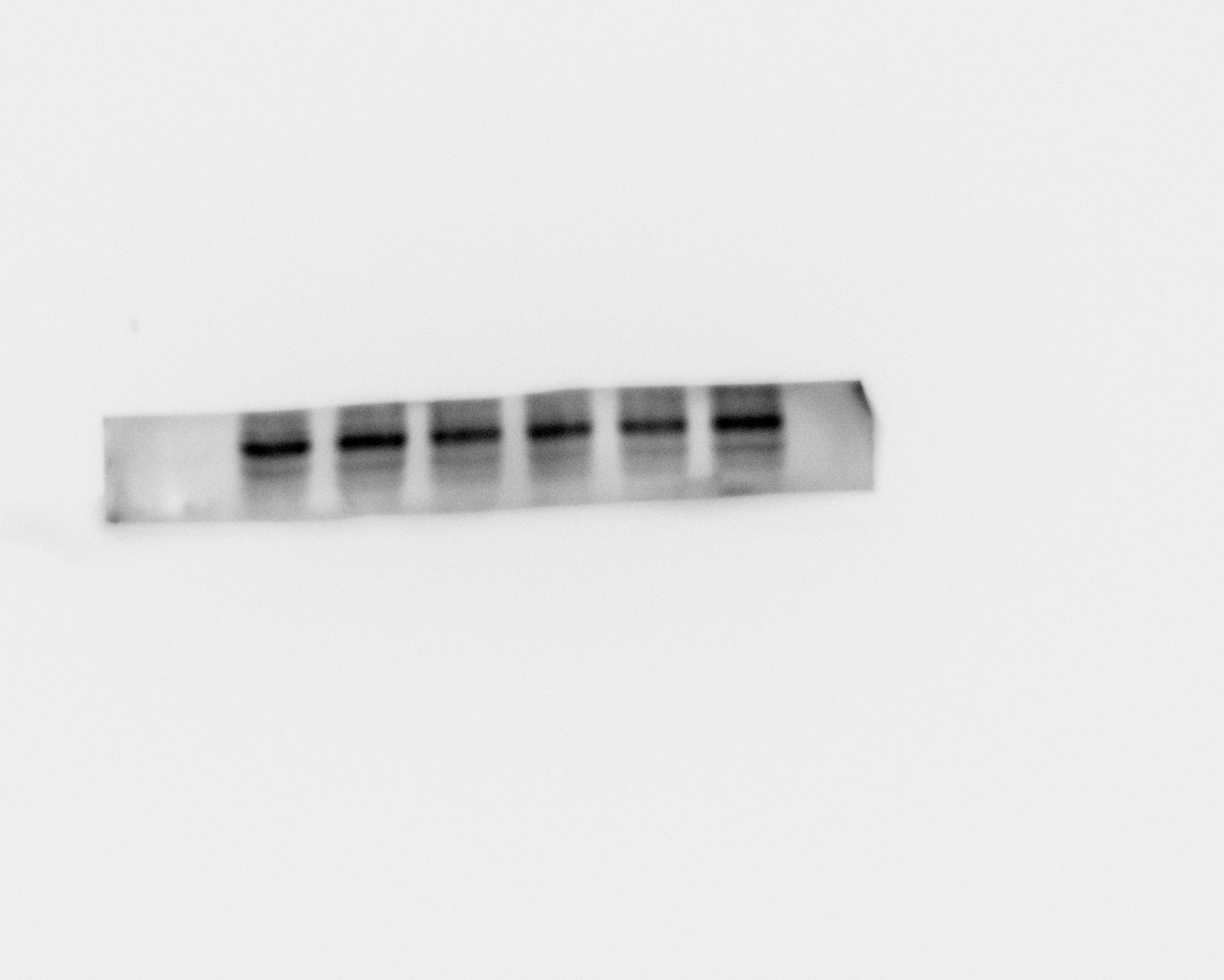

Supplement: Supplementary file 15 — Supplementary Material 15 [file 13058_2024_1864_MOESM15_ESM.tif]

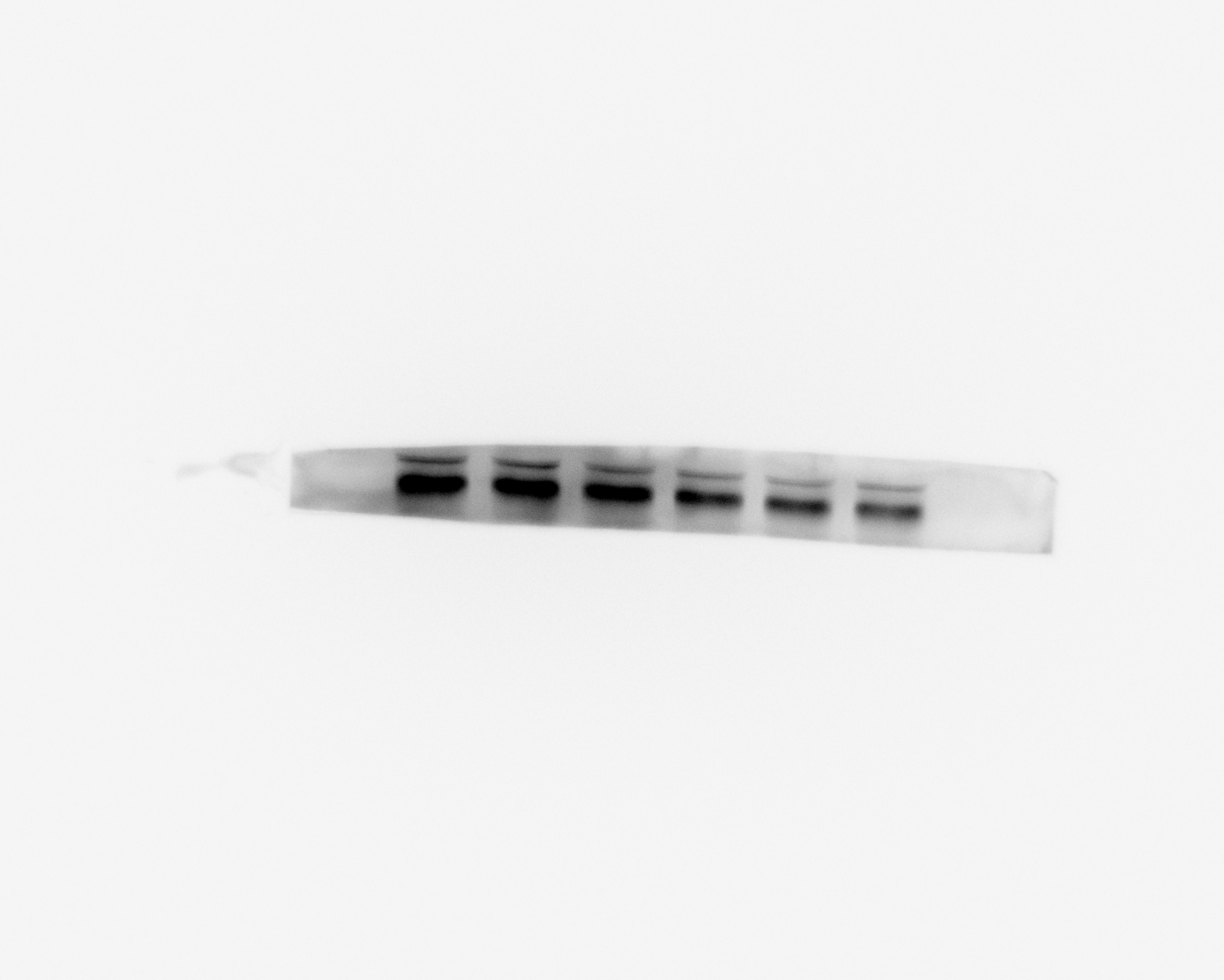

Supplement: Supplementary file 16 — Supplementary Material 16 [file 13058_2024_1864_MOESM16_ESM.tif]

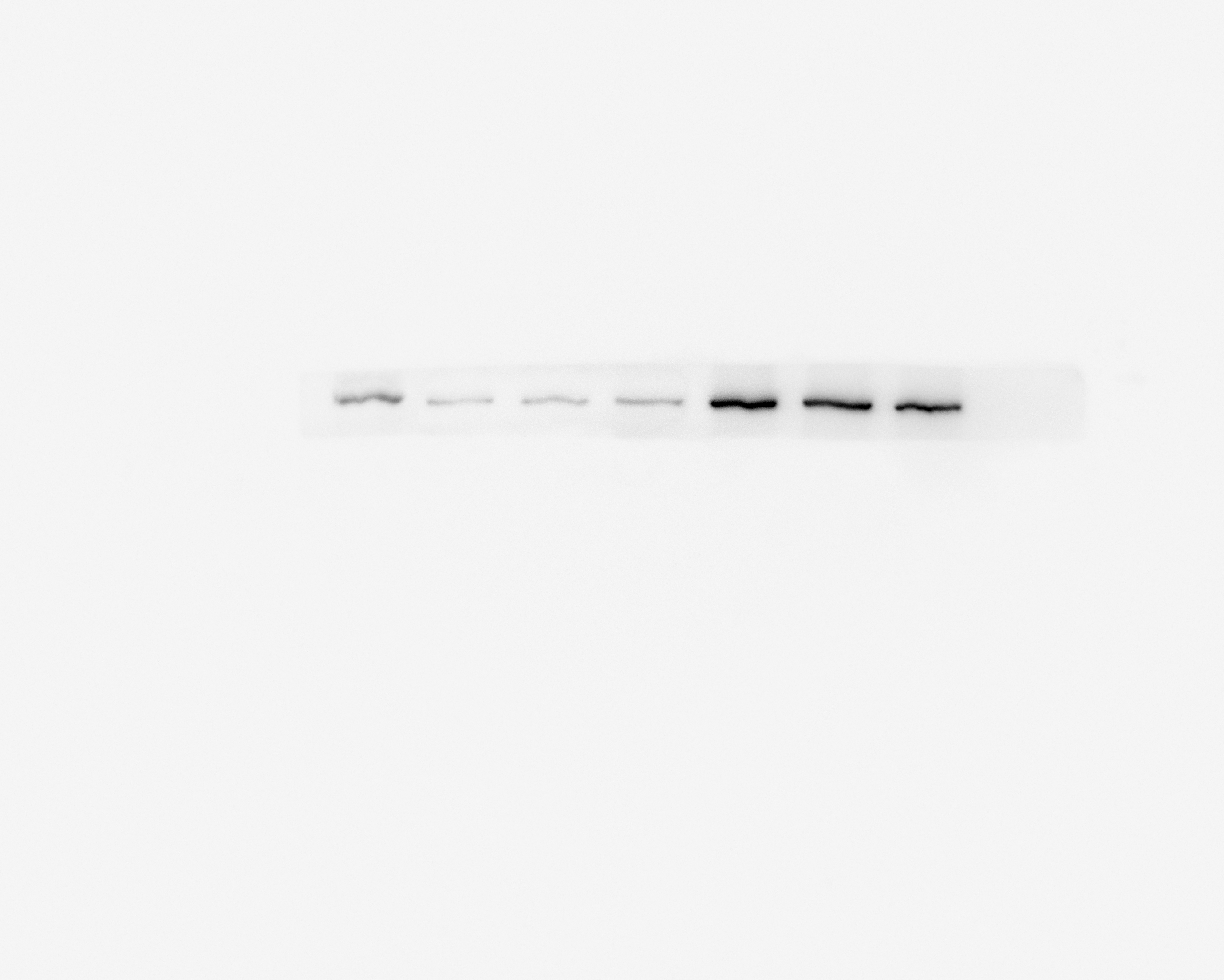

Supplement: Supplementary file 17 — Supplementary Material 17 [file 13058_2024_1864_MOESM17_ESM.tif]

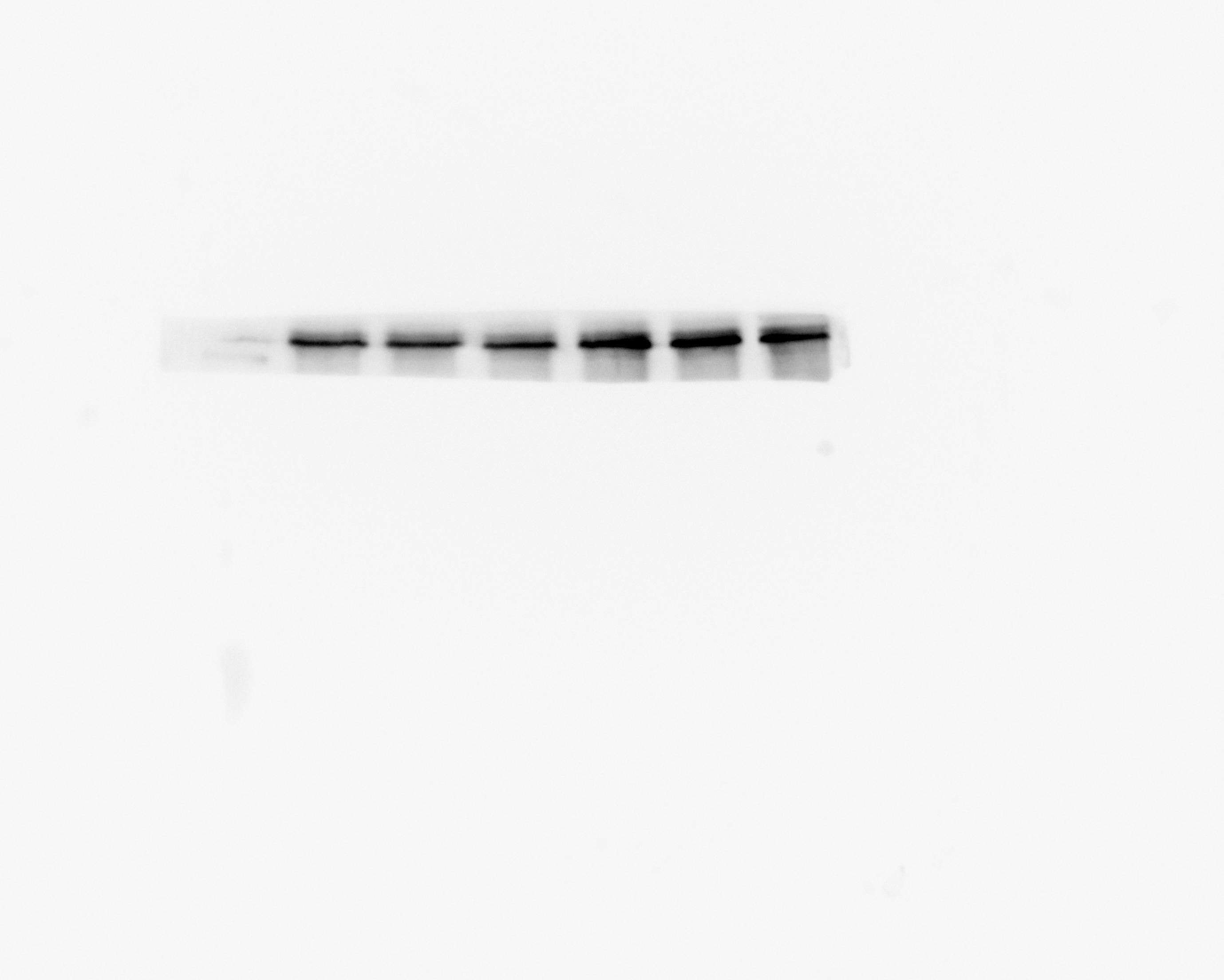

Supplement: Supplementary file 18 — Supplementary Material 18 [file 13058_2024_1864_MOESM18_ESM.tif]

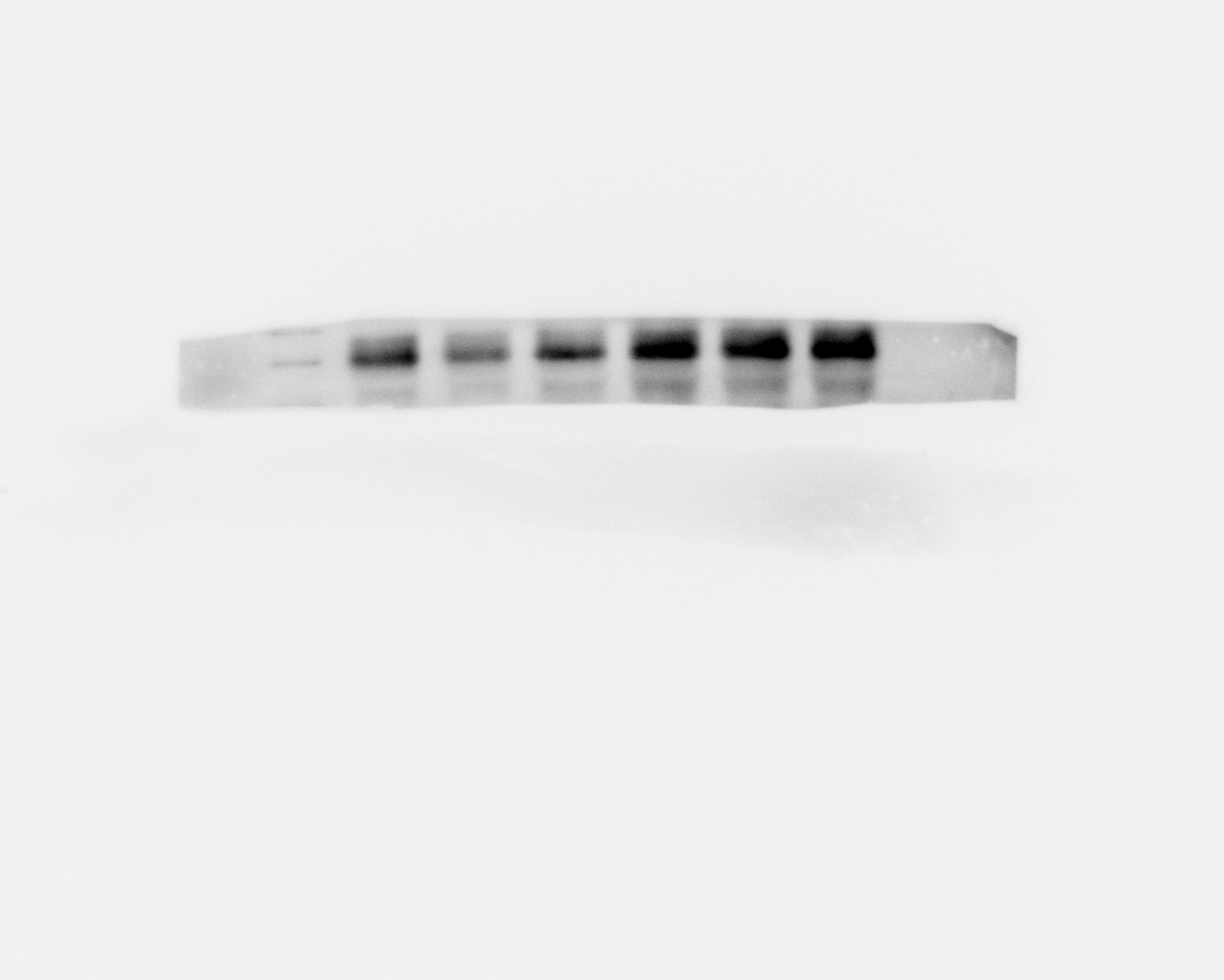

Supplement: Supplementary file 19 — Supplementary Material 19 [file 13058_2024_1864_MOESM19_ESM.tif]

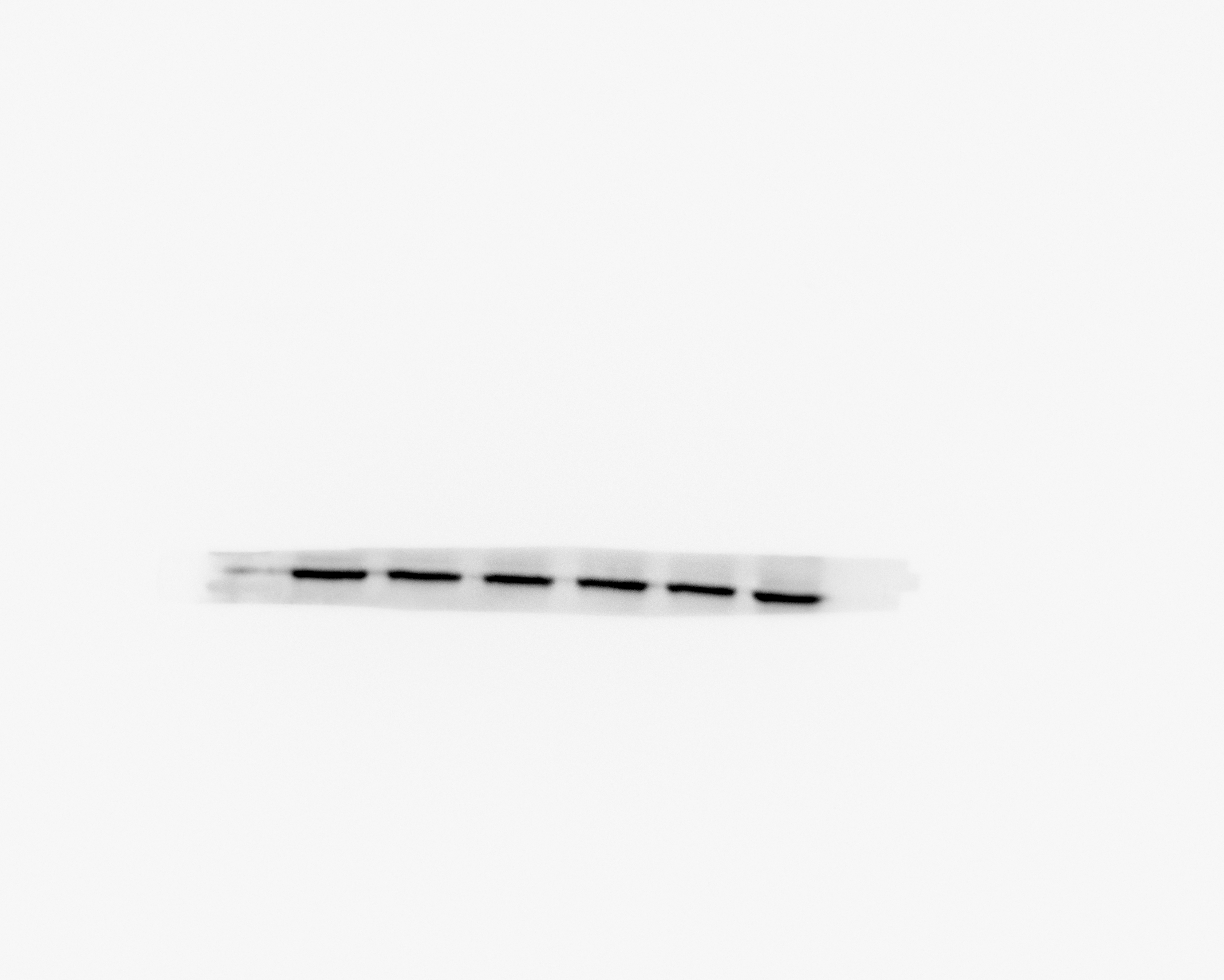

Supplement: Supplementary file 20 — Supplementary Material 20 [file 13058_2024_1864_MOESM20_ESM.tif]

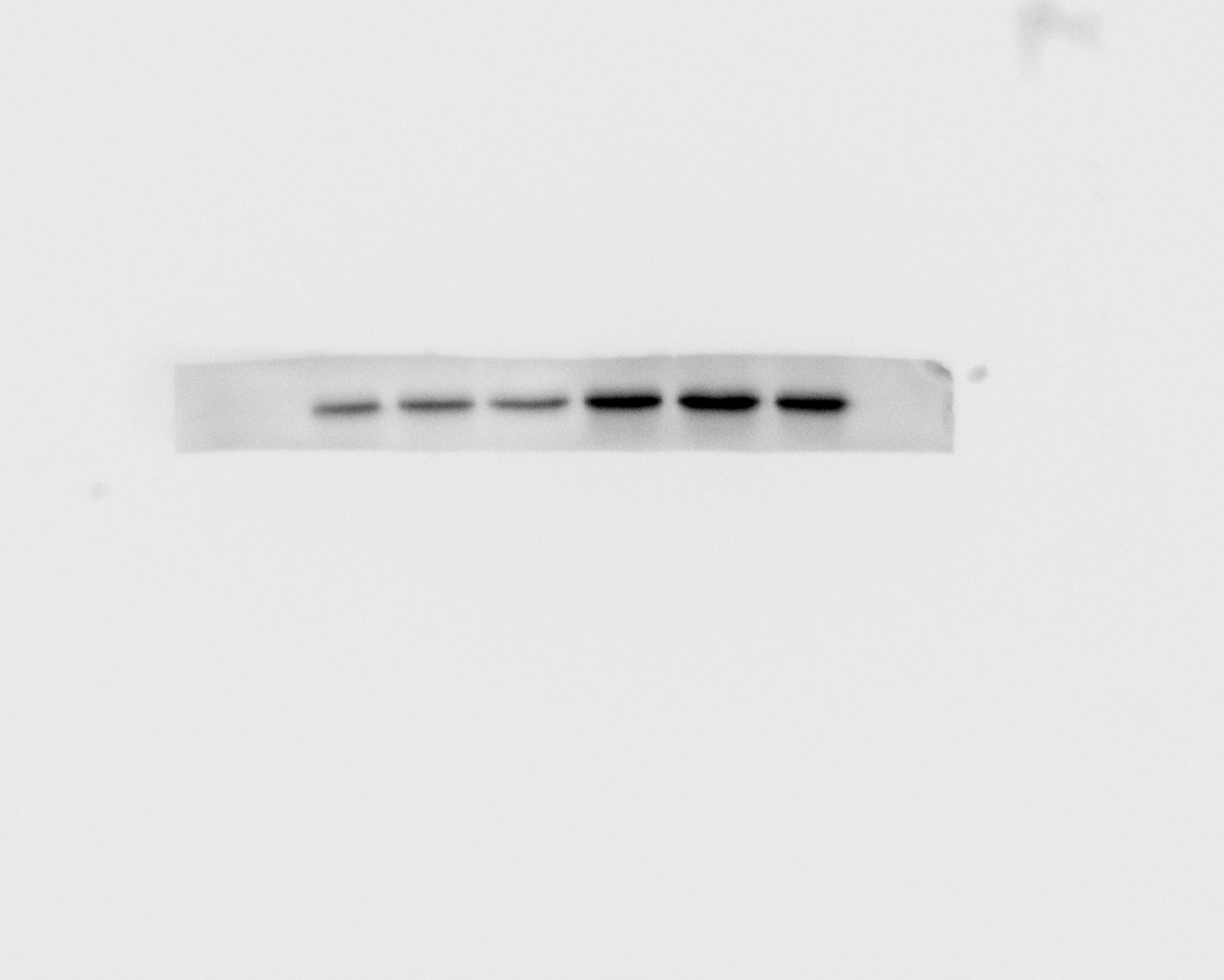

Supplement: Supplementary file 21 — Supplementary Material 21 [file 13058_2024_1864_MOESM21_ESM.tif]
